# Hyperuricemia and its related diseases: mechanisms and advances in therapy

**DOI:** 10.1038/s41392-024-01916-y

**Published:** 2024-08-28

**Authors:** Lin Du, Yao Zong, Haorui Li, Qiyue Wang, Lei Xie, Bo Yang, Yidan Pang, Changqing Zhang, Zhigang Zhong, Junjie Gao

**Affiliations:** 1 Sports Medicine Center, The First Affiliated Hospital of Shantou University Medical College, Shantou, 515041 China; 2 Institute of Sports Medicine, Shantou University Medical College, Shantou, 515041 China; 3 Centre for Orthopaedic Research, Medical School, The University of Western Australia, Nedlands, WA 6009 Australia; 4 Department of Orthopaedics, Shanghai Sixth People’s Hospital Affiliated to Shanghai Jiao Tong University School of Medicine, Shanghai, 200233 China

**Keywords:** Metabolic disorders, Therapeutics

## Abstract

Hyperuricemia, characterized by elevated levels of serum uric acid (SUA), is linked to a spectrum of commodities such as gout, cardiovascular diseases, renal disorders, metabolic syndrome, and diabetes, etc. Significantly impairing the quality of life for those affected, the prevalence of hyperuricemia is an upward trend globally, especially in most developed countries. UA possesses a multifaceted role, such as antioxidant, pro-oxidative, pro-inflammatory, nitric oxide modulating, anti-aging, and immune effects, which are significant in both physiological and pathological contexts. The equilibrium of circulating urate levels hinges on the interplay between production and excretion, a delicate balance orchestrated by urate transporter functions across various epithelial tissues and cell types. While existing research has identified hyperuricemia involvement in numerous biological processes and signaling pathways, the precise mechanisms connecting elevated UA levels to disease etiology remain to be fully elucidated. In addition, the influence of genetic susceptibilities and environmental determinants on hyperuricemia calls for a detailed and nuanced examination. This review compiles data from global epidemiological studies and clinical practices, exploring the physiological processes and the genetic foundations of urate transporters in depth. Furthermore, we uncover the complex mechanisms by which the UA induced inflammation influences metabolic processes in individuals with hyperuricemia and the association with its relative disease, offering a foundation for innovative therapeutic approaches and advanced pharmacological strategies.

## Introduction

Hyperuricemia is a metabolic disorder marked by elevated serum uric acid concentrations in both extracellular fluids and tissues, coupled with impaired uric acid excretion.^1^ The definition of hyperuricemia is SUA level ≥ 7.0 mg/dl (416.0 μmol/L) in males or ≥ 6.0 mg/dl (357.0 μmol/L) in females.^2^ Hyperuricemia is associated with various risk factors, including a high-purine diet, alcohol consumption, medication usage, hypertension, hypothyroidism, and obesity. Additionally, social factors such as higher socioeconomic status, as well as a history of smoking and alcohol use, further contribute to the heightened risk of developing this condition.^1,3,4^ UA plays a double-edged sword role in humans.^5^ Uric acid possesses antioxidant capabilities that combat free radicals and reactive oxygen species, thus preventing oxidative stress.^6–8^ The antioxidant effect of uric acid can be manifested in the inhibition of cell death to protect nerves as well as profile support of NO-mediated vasodilation.^9^ However, uric acid will be transformed into a pro-oxidant and pro-inflammatory molecule that exacerbates oxidative stress when the UA levels are increased.^10–12^ UA, mediates the innate immune response, which can release inflammatory mediators and activate the renin-angiotensin system,^13^ inflammatory responses, oxidative stress, vascular endothelial dysfunction and insulin resistance.^14–16^ Mendelian randomization studies have demonstrated no causal relationship between elevated uric acid levels and the risks of diabetes, coronary heart disease, ischemic stroke, heart failure, body mass index, bone mineral density, coronary artery disease, blood pressure, metabolic syndrome, blood glucose levels, triglyceride levels, diabetes mellitus, serum creatinine levels, glomerular filtration rate, and Parkinson’s disease.^17–21^ The only phenotypes that were causally associated with HU were gout and kidney disease.^22^ However, epidemiological and clinical studies have linked hyperuricemia to the development of various conditions, including chronic kidney disease, fatty liver, metabolic syndrome, hypertension, insulin resistance, obesity, type 2 diabetes, and cardiovascular and cerebrovascular disorders.^1,23–25^ In this article, we review the complex physiological roles and metabolism of uric acid and the interconnections of mechanisms between hyperuricemia and potential diseases. Furthermore, we summarize the novel therapeutic interventions for hyperuricemia by examining its common comorbidities, underlying mechanisms, phenotypes, and pathogenesis.

## The timescale and prevalence of hyperuricemia

**Fig. 1:**
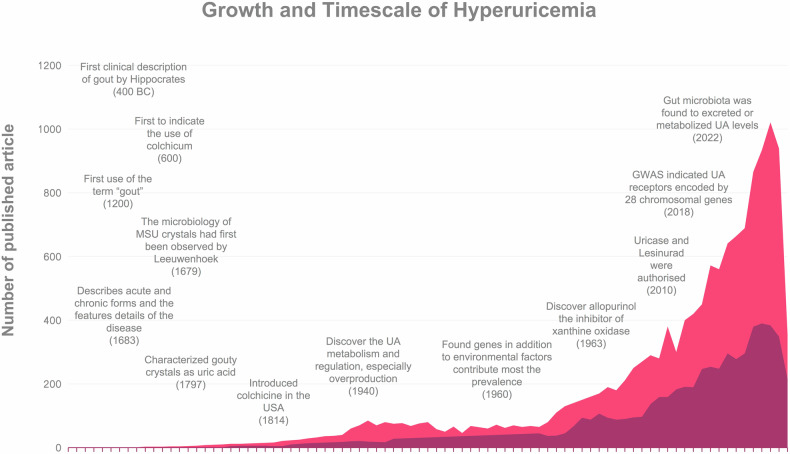
The timescale and historical development of hyperuricemia (depicted in light red) and hyperuricemia treatment (depicted in dark red) from 1944 to June 2024, along with the volume of published literature, have been analyzed using data extracted from PubMed. The search criteria included “hyperuricemia*“ in conjunction with terms such as “history”, “medicine”, “treatment”, “therapy”, “drug”, “mechanism”, “genetic”, and “uric acid”

Among history, Podagra first developed and identified by Hippocrates, which called “unwalkable illness” in 400 BC. with the definition podagra as a style called “arthritis of the rich.” Over 2000 years ago, Colchicine, initially used as a purgative in ancient Greece, which was later recognized by Alexander of Tralles in the sixth century AD for its specific therapeutic effects on arthritis.^26^ By 1200, gout was dubbed the ‘disease of kings’ due to its association with a luxurious lifestyle. In 1679, Antonie van Leeuwenhoek, a pioneer in microbiology, first observed crystals from tophi in gout patients. The chemical composition of uric acid was identified by a Swedish chemist in 1797, and by 1940, the understanding of uric acid metabolism, including its excretion and overproduction was established.^27–30^ The role of genetic factors in hyperuricemia prevalence was discovered in the 1960s. In 1963, the introduction of Allopurinol, an inhibitor of xanthine oxidase, marked a significant advancement in treating hyperuricemia. More recently, in 2010, uricase enzymes like Pegloticase and Rasburicase were approved for the management of persistent arthritis in patients with comorbidities and joint deformities.^31–33^ Currently, the emerging drugs and some advanced treatments such as uricosuric compounds, antidyslipidemic drugs or gut microbiota, can reduce the concentration of serum uric acid to address resistant hyperuricemia (Fig. 1).

Hyperuricemia is a globally prevalent condition, particularly in high- and middle-income countries. Its prevalence varies significantly due to factors such as geographic location, regional differences, ethnicity, dietary habits, and economic conditions. Recent trends indicate an increase in the prevalence of hyperuricemia.^2,3^ The global prevalence rate has been reported to be ranging from 2.6% to 36% in different populations.^34^ The U.S. National Health and Nutrition Examination Survey (NHANES) indicates that approximately 21% of adults, or 43 million individuals, have been diagnosed with hyperuricemia.^35^ Comparative prevalence rates are as follows: 16.6% in Australia,^36^ 48% in Finland with a gender-specific breakdown of 60% in males and 31% in females,^37^ 17.0% in New Zealand with 27.8% in males and 8.8% in females,^38^ 24.5% in Ireland with 25.0% in males and 24.1% in females,^39^ 9.9% in Croatia,^40^ 16.8% in Russia,^41^ 12.1% in Turkey with 19.0% in male and 5.8% in female,^42^ 21.2% in Qatar.^43^ Likewise, among developing countries, the Korea NHANES reported that the prevalence of hyperuricemia in Korea was 11.4% with 17.0% in male and 5.9% in female.^44^ In addition, it is 20.6% in Mexico,^45^ 17.2% in Niger with 25.0% in male and 13.7% in female,^46^ 71.6% in French Polynesia,^47^ 9.9% in Croatia,^40^ 44.6% in India,^48^ 28.1% in Jordan,^49^ 31.8% in sub-Saharan African,^50^ 10.6% in Thailand with 18.4% in male and 7.8% in female,^51^ 8.4% in Saudi Arabia^52^ and approximately 9.3% in Bangladesh.^53^ (Table 1) As expected, the prevalence of hyperuricemia found in our study is higher in most developed countries. Interestingly, the prevalence of hyperuricemia is higher in coastal areas and countries than in landlocked countries, especially for countries surrounded by sea and in developing. China, with its large population, exhibits significant demographic diversity and regional differences. The prevalence of hyperuricemia was 6.4% in Chinese adults according to a study covering 13 provinces.^2^ Geographically, the prevalence of hyperuricemia is highest in southern China (9.1%) and lowest in northern China (3.2%). The majority of affected individuals (71.0%) reside in urban areas, with a substantial proportion (44.7%) living in coastal cities. The prevalence is notably higher in urban regions (8.0%) compared to rural areas (5.0%). By 2014, the overall prevalence of hyperuricemia in mainland China had reached 13.3%,^54^ and this gradually increased to 17.7% in 2017. The prevalence was higher in the elderly population, and the rate was higher in male (23.5%) than in female (11.7%).^55^

**Table 1:** Diagnostic criteria and prevalence for hyperuricemia in each country/area

Country/area	Male	Female	General	Prevalence
United States	7.0 mg/dL (420 µmol/L)	6.0 mg/dL (360 µmol/L)	7.0 mg/dL (420 µmol/L)	21%^35^
Japan	7.0 mg/dL (420 µmol/L)	6.0 mg/dL (360 µmol/L)	/	30% in male and 3% in female^440^
United Kingdom	6.8 mg/dL (404 µmol/L)	6.0 mg/dL (360 µmol/L)	/	27.72% in male and 10.69% in female^336^
India	/	/	7.0 mg/dL (420 µmol/L)	44.6%^48^
European Union	6.8 mg/dL (404 µmol/L)	5.7 mg/dL (339 µmol/L)	/	11.9%–25.0% of the European population^336^
China	7.0 mg/dl (420.0 μmol/l)	6.0 mg/dl (360.0 μmol/l)	7.0 mg/dL (420 µmol/L)	17.7%^54^
Australia	/	/	7.0 mg/dL (420 µmol/L)	16.6%^36^
Finland	6.8 mg/dL (404 µmol/L)	5.7 mg/dL (339 µmol/L)	/	48.0% (60% in male and 31% in female)^37^
New Zealand	/	/	7.0 mg/dL (420 µmol/L)	17.0% (27.8% in male and 8.8% in female)^38^
Ireland	6.8 mg/dL (404 µmol/L)	5.7 mg/dL (339 µmol/L)	/	24.5% (25.0% in male and 24.1% in female)^39^
Croatia	6.8 mg/dL (404 µmol/L)	5.7 mg/dL (339 µmol/L)	/	9.9%^40^
Russia	/	/	7.0 mg/dL (420 µmol/L)	16.8%^41^
Turkey	7.0 mg/dl (420.0 μmol/l)	6.0 mg/dl (360.0 μmol/l)	7.0 mg/dL (420 µmol/L)	12.1% (19.0% in male and 5.8% in female)^42^
Qatar	/	/	7.0 mg/dL (420 µmol/L)	21.2%^43^
Korea	7.0 mg/dl (420.0 μmol/l)	6.0 mg/dl (360.0 μmol/l)	7.0 mg/dL (420 µmol/L)	11.4% (17.0% in male and 5.9% in female)^44^
Mexico	/	/	7.0 mg/dL (420 µmol/L)	20.6%^45^
Niger	/	/	7.0 mg/dL (420 µmol/L)	17.2%^46^
French Polynesia	/	/	6.0 mg/dL (360 µmol/L)	71.6%^47^
Jordan	/	/	7.0 mg/dL (420 µmol/L)	28.1%^49^
sub-Saharan African	/	/	6.0 mg/dL (360 µmol/L)	31.8%^50^
Thailand	7.0 mg/dl (420.0 μmol/l)	6.0 mg/dl (360.0 μmol/l)	7.0 mg/dL (420 µmol/L)	10.6% (18.4% in male and 7.8% in female)^51^
Saudi Arabia	7.0 mg/dL (420 µmol/L)	6.0 mg/dL (360 µmol/L)	7.0 mg/dL (420 µmol/L)	8.4%^52^
Bangladesh	7.0 mg/dl (420.0 μmol/l)	6.0 mg/dl (360.0 μmol/l)	7.0 mg/dL (420 µmol/L)	9.3%^53^

/, the country without an exact criteria

## Physiological role of uric acid

Uric acid is the final product of the catabolism of purine nucleotides. UA is a weak diprotic acid with one dis-sociable H^+^ at physiologic pH values. The concentrations of UA range from 3.5 to 7.2 mg/dL (210–430 μmol/L) in males and 2.6–6.0 mg/dL (155–360 μmol/L) in premenopausal females.^2,35^ In addition to its role as a byproduct of purine metabolism, uric acid is recognized for its multifaceted effects, which include antioxidant, pro-oxidant, pro-inflammatory, nitric oxide regulation, immune system interactions, and anti-aging properties.^7,56^

### Antioxidant and Pro-oxidant

Uric acid is a natural byproduct of purine metabolism, arising from the enzymatic degradation of hypoxanthine to xanthine, which is subsequently converted by xanthine oxidase.^57^ In the process in which uric acid is produced, ROS, particularly superoxide anions and hydrogen peroxide (H_2_O_2_), are generated as byproducts.^58–61^ Uric acid functions as a powerful antioxidant, effectively neutralizing singlet oxygen molecules, oxygen radicals, and peroxynitrite (ONOO-) molecules, due to its ability to provide electrons and act as a powerful reducing agent.^27–29,62–65^ It can easily provide a hydrogen atom to free radicals, thereby stabilizing them and preventing further oxidative damage.^66^ Therefore, uric acid has remarkable antioxidant properties that effectively combat oxidative stress induced by free radicals and reactive oxygen species (ROS).^7^ Free radicals are highly reactive entities that can cause oxidative stress and cellular damage and contribute to the development of various diseases.^67^ Nevertheless, uric acid has a highly reducing structure that effectively neutralizes free radicals and mitigates their harmful effects. Additionally, uric acid acts as an inhibitor of the oxidative chain reaction through a dual mechanism.^68,69^ It captures and neutralizes free radicals, forming stable intermediates and thereby impeding the transmission of the oxidative reaction.^66^ Furthermore, the complex formed by uric acid and free iron ions acts as a chelating agent, effectively inhibiting the formation of free radicals from iron ions and enhancing the antioxidant effect.^67^ Uric acid regulates the inflammatory response by inhibiting the production of inflammatory mediators, which significantly reduces the formation of free radicals.^57^ One of the most interesting aspects of the antioxidant function of uric acid is its potential role in neuroprotection.^60–62^ Uric acid and purines, including adenosine and adenosine triphosphate, have been implicated in regulating central nervous system functions such as convulsive threshold, memory, cognition, sleep, activity, appetite, mood, social interaction, drive, impulsivity, and intelligence.^70–72^ Some studies have found that patients with neurodegenerative diseases, like Parkinson’s disease, Alzheimer’s disease, and amyotrophic lateral sclerosis (ALS), tend to have lower serum uric acid levels, suggesting a potential neuroprotective effect of uric acid.^73^ Patients with major depression and anxiety disorders had lower plasma uric acid levels and increased UA levels after treatment, further suggesting that UA may have a neuroprotective effect.^70^ The antioxidant properties of UA and its ability to inhibit oxidative stress may attenuate inflammatory damage to the nervous system and contribute to the maintenance of neuron number and function by inhibiting programmed apoptosis of neuronal cells, which protects against excessive neuronal cell damage. The capacity of uric acid to neutralize reactive oxygen species (ROS) and shield neurons from oxidative damage may underlie its observed neuroprotective effects. Furthermore, the antioxidant properties of uric acid have significant implications for cardiovascular health.^66^ The ability of uric acid to scavenge ROS and reduce oxidative stress may have protective effects on the cardiovascular system. Studies illustrated that uric acid may indirectly support NO-mediated vasodilation by preventing nitric oxide degradation by superoxide radicals. This finding implies that uric acid may play a role in maintaining vascular health and regulating blood pressure.^74^ The antioxidant function of serum uric acid reflects the multifaceted and complex nature of its physiological role. The ability of uric acid to neutralize free radicals and protect against oxidative stress has implications for all aspects of health.^67,75^ Interestingly, a level of uric acid that is either too high or too low disrupts the delicate balance of oxidative stress regulation and may lead to excessive oxidative damage or impaired antioxidant defense. At higher intracellular concentrations, uric acid can function as a pro-oxidant molecule.^76^ Studies have shown that within various cell types, including vascular smooth muscle cells, endothelial cells, adipocytes, hepatocytes, pancreatic islet cells, and renal tubular cells, uric acid can activate NADPH oxidase, a crucial enzyme involved in the generation of reactive oxygen species.^57,76^ Moreover, in certain cell types, NADPH oxidase may translocate to the mitochondria, further exacerbating oxidative stress.^77,78^ The effects of soluble urate on mononuclear cells are multifaceted. Some studies suggest that priming peripheral blood mononuclear cells (PBMCs) with urate enhances the release of interleukin-1β (IL-1β) in response to lipopolysaccharide (LPS), indicating a potential pro-inflammatory effect.^79^ While it was found no significant effects of urate on IL-1β release, superoxide dismutase 2 (SOD2) gene transcription, or the total antioxidant capacity of the cell.^80^

### Pro-inflammatory

Uric acid acts as a danger signal, being naturally released by necrotic cells and subsequently initiating adaptive immune responses. Studies have indicated that uric acid crystals can engage with Toll-like receptors (TLRs), which are membrane-bound receptors integral to innate immunity, thereby inducing inflammation.^10,11,81,82^ Specifically, TLR-2, TLR-4, and the myeloid differentiation primary response protein 88 (MyD88) are crucial to the inflammatory reaction of macrophages to uric acid crystals. These crystals can directly interact with these receptors, initiating signal transduction pathways that ultimately activate NF-κB.^83–87^ NF-κB is a transcription factor responsible for improving the transcription of various inflammation-associated proteins, including pro-interleukin-1 (pro-IL-1), when secreted in the extracellular space.^88^ In recent years, studies have revealed that UA activates the TLR4-NLRP3 inflammatory complex, which is a multi-protein complex that plays a pivotal role in initiating the innate immune response to various danger signals, including MSU crystals. Upon recognition of MSU crystals, the NLRP3 inflammasome is activated, leading to the cleavage of pro-inflammatory cytokines, specifically interleukin-1β (IL-1β) and interleukin-18 (IL-18).^89–93^ These cytokines play a central role in orchestrating the inflammatory response by recruiting additional immune cells and amplifying the proinflammatory cascade.^57^ UA exerts its influence on the renin-angiotensin system through dual mechanisms involving the stimulation of plasma renin activity and renal renin expression. Additionally, UA contributes to the activation of the intrarenal angiotensin system.^94^ These immune inflammatory pathways, particularly those involving monocytes and macrophages, are upregulated in the presence of hyperuricemia.^95,96^ The pro-inflammatory function of uric acid is critical for revealing its role in various inflammatory conditions, such as gout, cardiovascular disease, and metabolic syndrome.^82^ UA was observed to reduce reactive oxygen species (ROS) and interleukin-6 (IL-6) production in macrophages while enhancing fatty acid oxidation (FAO) under inflammatory and hypoxic conditions in vitro.^95^ Although the antioxidant properties of uric acid have long been recognized, its pro-inflammatory effects complicate its physiological significance and clinical relevance.

### Nitric oxide regulation

Nitric oxide (NO) is a vital signaling molecule produced by endothelial nitric oxide synthase (eNOS) within endothelial cells. It serves as a powerful vasodilator, modulating blood pressure by inducing relaxation in the smooth muscle cells of blood vessel walls.^97^ Hyperuricemia, by inducing oxidative stress and inflammation, diminishes the expression of eNOS and the synthesis of NO, while elevating levels of inflammatory cytokines such as IL-6 and TNF-*α*, ultimately impairing endothelial function.^98,99^ In addition, NO is involved in inhibiting platelet aggregation, leukocyte adhesion, and inflammation. It also contributes to various signaling pathways that affect cardiac function, nerve conduction, and the immune response.^100^ The interaction between uric acid and NO is bidirectional. When concentrations are low, uric acid acts as a natural antioxidant that scavenges free radicals and prevents oxidative damage. Specifically, uric acid neutralizes peroxynitrite, a harmful molecule formed from the reaction between nitric oxide and superoxide radicals.^101,102^ This antioxidant effect of uric acid protects nitric oxide from degradation by superoxide radicals, thereby indirectly supporting nitric oxide bioavailability and potentially enhancing nitric oxide-mediated vasodilation.^61,103^ However, at higher concentrations, uric acid reduces NO bioavailability, impairs eNOS function, reduces NO production, and further exacerbates endothelial cell dysfunction. These complex interactions have important implications for cardiovascular health, renal function, and treatment of NO-related diseases.^97,104,105^ Uric acid has a protective effect against dementia and cognitive impairment related to senescence.^72,106–110^ UA endowed with hydrophilic antioxidant properties which can exert a protective influence against Alzheimer’s disease and Parkinson’s disease, while hyperuricemia could potentially worsen vascular dementia, encompassing conditions such as stroke and small vessel cerebrovascular disease.^111^

### Aging and Anti-aging effects

Uric acid can influence cellular activities, such as cell proliferation, by modulating EGF/EGFR bioactivity. Hyperuricemia can downregulate the expression of cell cycle proteins including D1, p-Rb, Ki67, and CDK4, inducing cellular senescence and consequently diminishing EGF/EGFR signaling. Increased levels of uric acid result in inflammation and oxidative stress, which serve as potential risk factors for cellular senescence, apoptosis, and disruptions in the cell cycle. Conversely, physiological concentrations of uric acid (5 mg/dl) exhibit anti-aging effects by enhancing growth factor activity in aging cells. However, at higher concentrations (10 mg/dl), uric acid promotes cellular senescence and downregulates EGF/EGFR signaling.^112^

### Immune system interaction effects

The interaction of uric acid with the immune system involves the formation of monosodium urate (MSU) crystals. These uric acid crystals activate pattern recognition receptors (PRRs), including Toll-like receptors (TLRs), NOD-like receptors, and the NLRP3 inflammasome.^113–116^ Activation of these receptors initiates an inflammatory signaling cascade resulting in the secretion of pro-inflammatory cytokines and chemokines. These needle-shaped crystals can accumulate in diverse tissues, particularly in joints, eliciting an innate immune response. Consequently, immune cells, notably neutrophils and macrophages, are recruited to sites of crystal deposition.^10^ Moreover, neutrophils can phagocytose uric acid crystals and release various inflammatory mediators, such as interleukin-1β and ROS, further activating inflammation and amplifying the local inflammatory response.^113^ Studies have shown that UA also affects both T-cell populations and regulatory T-cell populations.^117^ UA-induced inflammation leads to the recruitment and activation of effector T cells at the site of crystal deposition, thereby exacerbating local inflammation.^113,117^

## The physiology of Hyperuricemia

Hyperuricemia is characterized by an elevated level of uric acid in the bloodstream, often surpassing the normal physiological threshold. This metabolic state arises from a dysregulation between uric acid production and elimination, culminating in its accumulation in the bloodstream.^118^ The etiology of hyperuricemia is multifaceted and involves genetic predispositions, environmental factors, and complex metabolic pathways governing urate homeostasis.

### Factors influencing uric acid

**Fig. 2:**
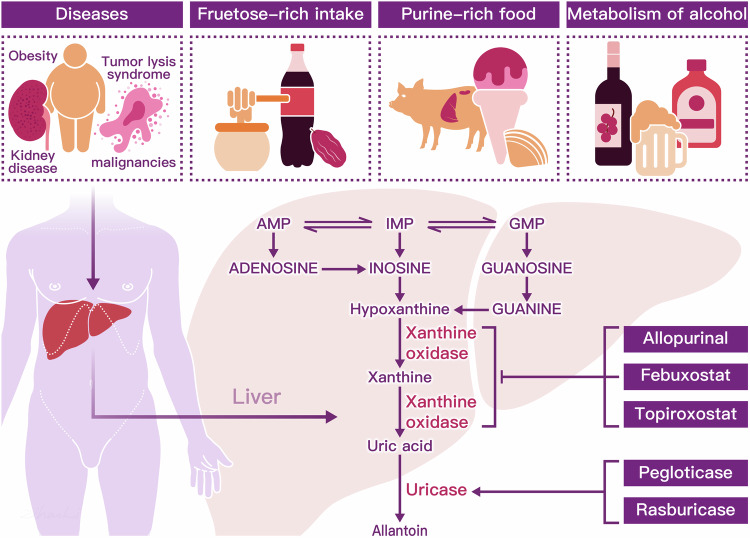
Consumption of purine-rich meats such as beef, pork, lamb, and seafood like oysters, shrimp, and tuna, as well as dietary fructose, are known to elevate uric acid (UA) production. Additionally, alcohol metabolism from beer and distilled spirits, along with certain medical conditions such as tumor lysis syndrome and obesity, pose increased risks for hyperuricemia. Hepatic metabolism of uric acid involves the sequential processing of purine nucleotides, including adenosine monophosphate (AMP), guanosine monophosphate (GMP), and inosine monophosphate (IMP).^66^ IMP plays a pivotal role as a key intermediate in purine nucleotide biosynthesis, serving as a precursor for the synthesis of both adenosine monophosphate (AMP) and guanosine monophosphate (GMP). Moreover, IMP can be enzymatically deaminated by IMP dehydrogenase, leading to the formation of inosine. Inosine, in turn, can undergo phosphorylation to become hypoxanthine. Hypoxanthine undergoes oxidative reactions catalyzed by xanthine oxidase (XOD), resulting in the production of xanthine. Xanthine can further undergo oxidation reactions, also catalyzed by XOD, ultimately leading to the formation of uric acid from xanthine.^118^ However, xanthine oxidase inhibitors, such as allopurinol, febuxostat, and topiroxostat, serve as first-line therapies by effectively reducing the production of uric acid from both endogenous and dietary purine sources. In the final step of purine metabolism, the enzyme uricase converts uric acid into allantoin, a highly soluble compound. While humans lack the uricase enzyme, animals naturally possess it. The therapeutic agents pegloticase and rasburicase are recombinant forms of uricase, designed to facilitate the breakdown of uric acid in humans

The risk of developing hyperuricemia is influenced by a combination of inherited genetic variants, environmental factors, gene-environment interactions, and intrinsic factors such as age, sex, and body weight.^119^ Research indicates that factors such as age, diet, alcohol consumption, fructose-rich intake, pharmacologic interventions and diseases, such as obesity, insulin resistance, Down syndrome, and kidney disease, contribute to the development of hyperuricemia^120–124^ (Fig. 2).

#### Dietary

Dietary selections abundant in purine, particularly nucleic acids, notably contribute to the production of uric acid. Beverages like beer, which contains purine-rich yeast, along with the consumption of foods such as bacon, beef, lamb, turkey, veal, venison, organ meats, and certain types of fish and shellfish (including anchovies, cod, tuna, sardines, shrimp, scallops, trout, and haddock), are implicated in elevating uric acid levels.^101,125^ Beer contains high amounts of guanosine, and ethanol increases the degradation of ATP. Alcohol and dietary purines (meat, seafood) may be risk factors for gout, which has traditionally been considered a disease of affluence. Sugar (sucrose) is a disaccharide composed of glucose and fructose.^126^ Among middle-aged Chinese men, a direct and notable association exists between seafood consumption and the occurrence of hyperuricemia. Conversely, protein intake from either animal or plant sources demonstrated a contrasting impact on the prevalence of hyperuricemia. Nevertheless, comprehensive data on the precise purine content of foods remains limited, primarily due to various factors such as food processing techniques, which can influence purine levels.^121^ According to the NHANES, dietary folate intake reduces the risk of hyperuricemia in female, while vitamin B12 and folate intake are associated with a reduced risk of hyperuricemia in men.^127^

#### Fructose metabolism

Fructose metabolism, particularly through the aldolase reductase pathway in the liver, results in increased UA levels.^128^ Fructose is transported into cells via SLC2A5 (Glut5) and undergoes metabolism to fructose 1-phosphate catalyzed by ketohexokinase (KHK), a process requiring ATP. This metabolic pathway primarily occurs in the liver, leading to a transient reduction in intracellular ATP and phosphate levels. Subsequently, activation of adenosine monophosphate (AMP) deaminase occurs, with AMP generated from fructose metabolism entering the purine catabolic pathway, ultimately resulting in the production of uric acid.^129^

#### Purine metabolism

Uric acid originates from the breakdown of purines catalyzed by the oxidized and reduced forms of xanthine oxidoreductase (XOR) and xanthine dehydrogenase (XDH). Purines are essential nucleotide components of DNA and RNA, crucial for cell division and metabolism.^101^ Both endogenous purine metabolism and dietary intake contribute to uric acid production. Increased cellular catabolism, heightened endogenous purine synthesis, and a diet rich in purines can elevate urate levels. Although basal XOR expression is typically low in humans, various factors such as hypoxia, ischemia-reperfusion injury, interleukin-1 (IL-1), interleukin-6 (IL-6), tumor necrosis factor-alpha (TNF-α), and corticosteroid treatment can markedly enhance XOR transcription. Additionally, the conversion of XDH to XO is expedited under hypoxic conditions.^101^ Purine metabolism occurs primarily in the liver and in tissues where xanthine oxidase is widely distributed. Approximately 65% of uric acid is excreted from the kidneys, and the rest is excreted mainly into the intestine.^130^ Due to the absence of the uricase enzyme responsible for converting uric acid into allantoin and allantoic acid, UA remains the terminal metabolic product in humans. The majority of uric acid is filtered in its free form, with approximately 90% of the filtered UA being reabsorbed.^131^ Hyperuricemia can be triggered by inadequate excretion due to reduced glomerular filtration, impaired tubular secretion and improved tubular reabsorption.^39,40^ Elevated phosphoribosyl pyrophosphate (PRPP) synthetase activity and deficiency in hypoxanthine phosphoribosyl transferase (HPRT) not only enhance endogenous purine synthesis but also result in excessive production and buildup of uric acid.^132,133^

#### Disease

Cellular turnover processes like tumor lysis, rhabdomyolysis, and hemolysis contribute to increased urate production. Additionally, various transporters in the intestinal mucosa and salivary glands, diverse medications, extracellular fluid volume depletion, and organic acids that facilitate transport can influence uric acid metabolism. Thus, both intrinsic and extrinsic factors play roles in urate production.^134^

### Uric acid regulation

UA levels are contingent on the dynamic equilibrium among purine-rich food intake, endogenous urate synthesis, and urate excretion through various routes, including urine and the gastrointestinal tract. Disruptions to this balance can impact serum uric acid (SUA) levels.^135–137^ The transport of uric acid entails multiple genes and proteins, collectively participating in the complex mechanisms of uric acid reabsorption and secretion. At a genome-wide significant level, three loci (ABCG2, SLC2A9, and CUX2) have been identified in association with renal urate overload, whereas four loci (ABCG2, SLC2A9, CUX2, and GCKR) have been linked to renal urate underexcretion.^138,139^ The main transporter genes are SLC22A12 (URAT1), SLC2A9 (GLUT9), and ABCG2 (BCRP).^140^(Table 2) It is increasingly recognized that disturbances in urate transport, both in the gastrointestinal tract and kidneys, are pivotal in the pathogenesis of diseases associated with hyperuricemia. Investigating these transporters and their genetic loci is essential for regulating and achieving target serum urate levels. Moreover, alterations in gut microbiota structure or imbalance can contribute to metabolic disorders, influencing the synthesis of purine-metabolizing enzymes and the release of inflammatory cytokines. This relationship is closely linked to the onset and progression of hyperuricemia and gout, which are metabolic immune disorders.^141,142^

**Table 2:** Urate transporters and their characteristics related to launched therapies for hyperuricemia

Transporter	Function	Location	Inhibitors
SLC22A12 (URAT1)	Mediating the reabsorption of UA from the renal tubular fluid back into the blood.	Apical membrane of the proximal tubule cells	Lesinurad; Benzbromarone; Arhalofenate (MBX201); Dotinurad; Tranilast
SLC2A9 (GLUT9)	Mediating the transport of urate from the tubular cells back into circulation, influencing renal urate reabsorption.	Apical and basolateral membrane of the proximal tubule	Benzbromarone; Tranilast
ABCG2 (BCRP)	Secretion of urate into the tubular lumen, facilitating renal urate excretion.	Apical membrane of renal tubules and intestine epithelial cells	Topiroxostat
SLC22A6 (OAT1)	Uptake of urate from the interstitial space into the tubular cells, contributing to urate secretion.	Basolateral side of the proximal tubule	Probenecid
SLC22A8 (OAT3)	Uptake of urate into renal tubular cells, facilitating urate secretion.	Basolateral membrane of the proximal tubule	Probenecid
SLC22A11 (OAT4)	Apical uptake of urate into renal tubular cells, potentially participating in urate reabsorption.	Apical side of the proximal tubule	Lesinurad; Arhalofenate (MBX201)
SLC17A1 (NPT1)	Secretion of urate into the tubular lumen, influencing renal urate excretion.	Apical membrane of the proximal tubule	
SLC17A3 (NPT4)	Secretion of urate into the tubular lumen, contributing to renal urate excretion.	Apical side of the renal proximal tubule	
PDZK1	PDZK1 acts as a scaffold protein, regulating the activity of various transport proteins in the proximal tubules, including URAT1 and NPT1. It enhances the UA reabsorption capacity of URAT1 and may influence the function of ABCG2.	Apical membrane of the proximal tubular in kidney	

#### Gut microbiota

UA serves as both an antioxidant and an immune modulator, exerting significant influence on the composition of the gut microbiota. Notably, the gastrointestinal tract plays a pivotal role as a pathway for uric acid excretion, with the microbial ecosystem within the gut intricately involved in this metabolic process.^143^ Transporters for uric acid located in intestinal epithelial cells facilitate the translocation of uric acid from the bloodstream into the intestinal lumen.^144–152^ Once in the intestinal lumen, UA can either be directly excreted or metabolized by the gut microbiota.^153^ Specific bacteria, such as Lactobacillus and Pseudomonas, participate in the degradation and elimination of uric acid in the intestine through the production of short-chain fatty acids (SCFAs).^154^ Moreover, the activities of enzymes involved in uric acid metabolism are intricately connected to the gut microbiota.^155–157^ Uricase, an enzyme responsible for converting UA into allantoin and urea, is found in various bacterial species including Bacillus pasteurii, Proteus mirabilis, and E. coli. Certain strains of Lactobacillus, such as Lactobacillus sp. OL-5, Lactobacillus plantarum Mut-7, and Lactobacillus plantarum Dad-13, have been found to exhibit higher intracellular uricase activity, further emphasizing the role of gut microbiota in UA metabolism.^143,149,154^ An imbalance in the gut microbiota can elevate uric acid concentrations, thereby exacerbating the chronic deposition of UA crystals in joints, characteristic of gout. This dysbiosis typically involves a proliferation of opportunistic pathogens alongside a reduction in beneficial bacteria known to stimulate the synthesis of anti-inflammatory cytokines.^145,158,159^ The exploration of intestinal flora metabolism represents a promising frontier in clinical research pertaining to hyperuricemia and gout. Metabolic research has established a correlation between hyperuricemia and disruptions in the primary bile acid pathway or intestinal metabolism, suggested that targeting the gut microbiome could offer innovative therapeutic approaches for managing hyperuricemia and its associated complications. Future studies will concentrate on unraveling the intricate mechanisms through which gut microbiota modulates metabolic processes in these patient populations.

#### Genetics of urate control

**Fig. 3:**
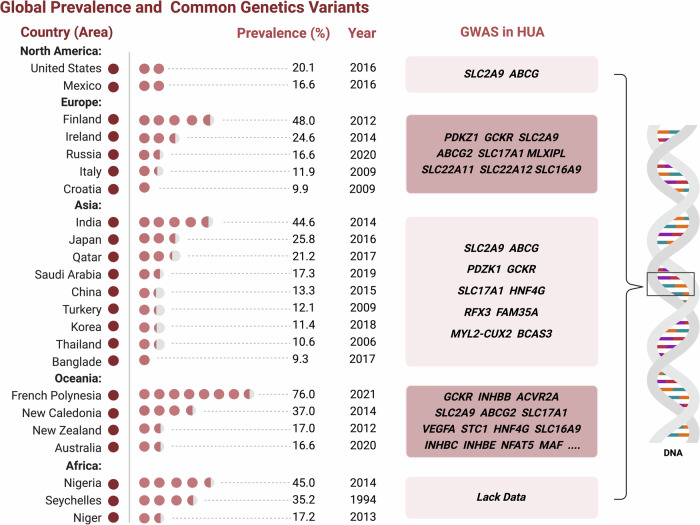
In East Asian populations, four loci have demonstrated a significant association with serum urate levels: SLC2A9, ABCG2, SLC22A12, and MAF. Similarly, in African American populations, three loci have been identified: SLC2A9, SLC22A12, and SLC2A12. In contrast, the European population predominantly shows an association with only one locus, SLC2A9. Australian studies have identified 28 loci, encompassing all but one (SLC2A12) of those found in African American and East Asian populations. Among these diverse populations, certain loci, such as SLC2A9, ABCG2, GCKR, and SLC17A1-SLC17A4 (also known as NPT1-NPT4), exhibit stronger effects and have been consistently replicated in multiple studies

Hyperuricemia and hyperuricosuria have been shown to cluster in families, indicating a familial transmission pattern. Studies in South American ethnic groups have demonstrated heritability rates ranging from 39% to 45%.^160–165^ Both adults and children have been found to exhibit genetic mutations that affect baseline renal urate excretion levels. In a genome-wide association study (GWAS) involving more than 140,000 individuals of European descent, uric acid receptors encoded by twenty-eight chromosomal genes were discovered to impact plasma uric acid levels. Genetic variants associated with uric acid levels mainly include purine metabolism (e.g., XDH, HPRT1), urate transporters (e.g., SLC2A9, SLC22A12), and renal urate processing regulators (e.g., ABCG2).^119,166,167^ Single nucleotide polymorphisms (SNPs) within or near these genes have consistently shown associations with variations in uric acid levels across diverse populations.^168^ Genome-wide association studies (GWAS) have identified key loci housing urate transporters crucial for uric acid excretion in both renal and gastrointestinal pathways.^167,169^ Through functional insights and expression quantitative trait loci (eQTL) analyses, several loci have identified probable causal genes, such as SLC2A9, ABCG2, PDKZ1, SLC22A11 (OAT4), and INHBB.^166^ Additionally, numerous other loci have strong candidate genes identified, including GCKR, RREB1, SLC17A1 to SLC17A4, SLC22A12, MAF, MLXIPL, PRKAG2, HNF4G, A1CF, IGFR1, and HLF.^166,170^ (Fig. 3) The primary physiological regulation of serum uric acid levels occurs through renal excretion.^171^ GWAS in major populations consistently highlight urate transporter genes as pivotal loci influencing serum uric acid levels,^138,139^ notably SLC2A9 (GLUT9) and SLC22A12 (URAT1), involved in urate reabsorption from urinary filtrates.^172,173^ For instance, the primary effect of SLC2A9 (rs12498742) explains 2% to 3% of serum uric acid level variance in Europeans, which is substantial for a complex phenotype.^174,175^ Variation in ABCG2 (BCRP) is also noteworthy across European and East Asian populations.^176–178^ Notably, in individuals of European ancestry, the genetic control of SLC2A9a and SLC2A9b isoforms, situated at basolateral and apical membranes respectively, constitutes a prominent genetic signal.^172,174^ Thus, we concluded several key genes have been identified with significant associations with SUA levels. Among these genes, ABCG2 stands out as one of the most crucial and strongly linked to SU levels to the risk of hyperuricemia.^167,171,179,180^ A specific polymorphism (rs2231142) within ABCG2 has been identified, which reduces urate efflux activity and increases the susceptibility to both HU and gout.^181^ Notably, this variant is more prevalent in Asian populations compared to Europeans.^180,182^ Another important gene, SLC2A9, encodes a urate transporter and exerts a significant influence on SUA levels. A specific polymorphism (rs734553) within SLC2A9 has been associated with an elevated risk of hyperuricemia.^119,166^ Interestingly, the prevalence of this variant varies among different populations, with higher frequencies observed in Asian populations. Research indicates that the rs1967017 variant in PDZK1 creates a binding site for the transcription factor hepatocyte nuclear factor 4α (HNF4α) within an enhancer region upstream of the PDZK1 transcription start site.^170^ This binding increases PDZK1 expression, potentially leading to reduced urate excretion. Another prominent genetic variant associated with serum urate levels is rs1263026 at GCKR.^183^ The Leu allele of this variant induces relaxation of glucokinase inhibition, resulting in heightened glucose phosphorylation. This process diminishes the ATP pool and augments urate production through ADP catabolism.^184^ However, other loci with more modest effects have not consistently replicated in subsequent studies examining their correlation with serum urate levels.

##### URAT1

URAT1, located in the membrane of renal tubular epithelial cells, serves as a uric acid transporter protein. It plays a crucial role in mediating UA reabsorption, a process in which approximately 90% of uric acid is typically reabsorbed following glomerular filtration.^175^ URAT1 belongs to the organic anion transporter (OAT) subgroup within the broader gene family. Other subcategories within this family include organic cation transporters, as well as novel type/carnitine transporters. Moreover, additional genetic alterations in hyperuricemia and gout associated with PDZK1 likely occurs through its modulation of the apical membrane localization of URAT1.^55^ Research has shown that individuals with renal hypouricemia and loss-of-function mutations in URAT1 demonstrate incomplete responses to both pyrazinamide and uricosurics, resulting in average concentrations reaching 0.93 mg/dL.^185^

##### GLUT9

GLUT9 functions as the principal transporter for urate efflux across the basolateral membrane of the proximal tubule in the kidney, facilitating transepithelial urate absorption.^186^ The pronounced lack of renal reabsorption of filtered urate in hypouricemic patients with GLUT9 loss-of-function mutations provides compelling evidence of the critical role this protein plays in renal tubular urate reabsorption. In these individuals, the fractional excretion of urate approaches 150%, highlighting the predominant mechanism of tubular urate secretion in the absence of reabsorption.^134^

##### BCRP

BCRP is an efflux pump that is driven by ATP on the apical membrane proximal renal tubule and intestinal epithelial cells and is critical for UA excretion. Mutated or dysfunctional ABCG2 may lead to significantly reduced excretion, moderate hyperuricemia and metabolic syndrome.^139^ Initially, it was hypothesized that the loss or reduction of ABCG2-mediated renal urate secretion would result in increased renal urate reabsorption, as diminished renal excretion is typically considered the primary mechanism of hyperuricemia in most gout patients. However, hyperuricemic patients with varying degrees of ABCG2 dysfunction, categorized by genotypes of dysfunctional SNPs, exhibit hyperuricemia characterized by urate overproduction. This is evidenced by elevated urinary urate excretion and a fractional excretion exceeding 5.5%. Additionally, ABCG2 dysfunction appears to contribute to renal underexcretion of urate in patients with milder functional impairments, also classified by genotype.^136^

##### OAT1, OAT3 and OAT4

OAT1 and OAT3, located on the basolateral membrane of the proximal tubule, function as urate/dicarboxylate exchangers responsible for uric acid excretion. Additionally, OAT4 participates in the transport of high-affinity binding steroids such as estrone sulfate (ES).^187^ This transporter operates as a chloride-ion-dependent exchanger for both ES and uric acid. Physiologically, OAT4 facilitates uric acid excretion in the proximal tubule by orchestrating ion exchange processes such as PAH/Cl-, PAH/ES, and potentially PAH/UA interactions. Its interplay with NHE3 and sodium dicarboxylate transporter 1 contributes to the regulation of intracellular α-ketoglutarate levels.^134^

##### NPT1 and NPT4

NPT1, which exhibits a weak to moderate correlation with altered uric acid levels, facilitates both the absorption and efflux of urate. It functions as a chloride-dependent urate transporter, which is involved in sodium/phosphate cotransport activities.^188^ NPT4 is crucial in urate excretion, working synergistically with basolateral organic anion transporters 1 and 3 (OAT1/OAT3). Uric acid was absorbed by OAT1 and OAT3 into tubular cells, which is subsequently transported into the urinary lumen by NPT4.^189^

##### PDZK

Polyvalent PDZ domain 1 (PDZK1) is a multidomain protein with four PDZ domains, primarily located at the apical membrane of kidney proximal tubule cells. It is abundantly expressed in this region and engages directly with several apical transporters, such as URAT1 and NPT1.^134^ As a scaffold protein, PDZK1 significantly regulates the activity of various transport proteins within the proximal tubules. Additionally, PDZK1 is proposed as a potential upstream regulator of ABCG2, impacting its function in the small intestine. Specifically, the upregulation of ABCG2 expression and function in response to soluble uric acid in intestinal cell lines is dependent on PDZK1 at the transcriptional level.^188^

**Fig. 4:**
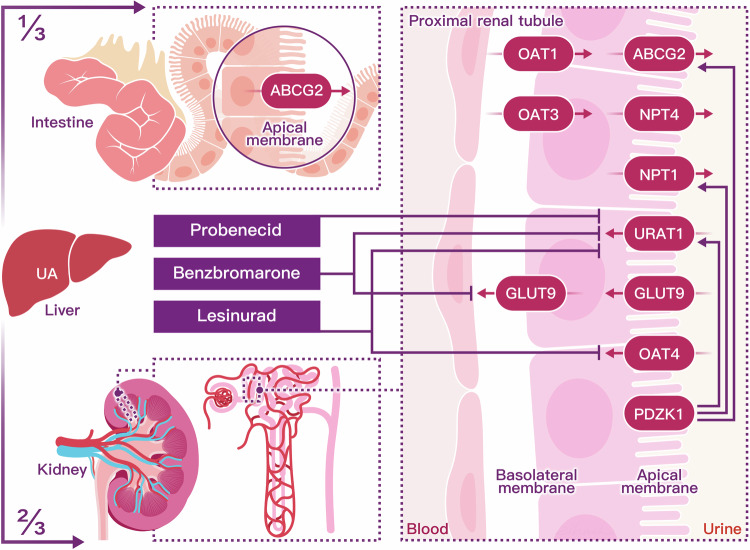
Uric acid undergoes a dynamic process of elimination and reabsorption, primarily orchestrated by the kidneys (two-thirds) and the intestines (one-third). In the nephron, filtration of water and solutes occurs within the glomerular capsule, followed by tubular reabsorption, predominantly mediated by the proximal convoluted tubule. Concurrently, tubular secretions extract uric acid from peritubular capillaries, secreting it into the tubular fluid for urinary excretion. Urate transporters in renal proximal tubule epithelial cells actively mediate the secretion and reabsorption of urate, thus determining the net excretion levels from the kidney. In the renal proximal tubule, SLC22A12 (URAT1), SLC17A1 (NPT1), and SLC22A11 (OAT4) located on the apical membrane facilitate reabsorption. SLC2A9 (GLUT9), found in both the apical and basolateral membrane tubules, is a long isoform that mediates the basolateral efflux of urate back into circulation. For excretion, SLC22A6 (OAT1) and SLC22A8 (OAT3) on the basolateral membrane facilitate urate entry into the renal tubules. ABCG2 (BCRP) and SLC17A3 (NPT4), positioned on the apical side, contribute to the secretory transport of urate into the tubule lumen for urinary excretion. In intestinal metabolism, uric acid is actively secreted into the intestinal lumen primarily by the transporter ABCG2, underscoring the role of the intestines in urate homeostasis

These genes are specifically expressed on the apical membrane of renal proximal tubule cells, which are crucial for the secretion of uric acid into the glomerular filtrate, as depicted in Fig. 4. Beyond the genes that encode for these transporter proteins, over a hundred genetic loci have been associated with hyperuricemia. Genome-wide association studies provide a comprehensive and unbiased method for pinpointing genetic factors linked to urate regulation and metabolism.^190^

## Hyperuricemia and diseases

An elevated uric acid concentration above physiological levels can pose a potential risk factor for several diseases closely associated with metabolic disorders. Numerous epidemiological studies have suggested that hyperuricemia may correlate with hypertension, metabolic syndrome, insulin resistance, dyslipidemia, type II diabetes, kidney disease, and cardiovascular events including coronary heart disease and cerebrovascular disease.^191–199^ Studies have demonstrated that serum uric acid levels can also predict the onset of hypertension, diabetes, obesity, and renal disorders^74,200,201^ (Table 3).

**Table 3:** The mechanisms of conditions caused by hyperuricemia

Diseases	Symptoms	Mechanisms related to hyperuricemia
Gout	Arthritis Tophi deposits and loss of mobility Destruction of cartilage and bone	NLRP3 inflammasome, orchestrating the inflammatory cascade in response to MSU crystal deposition Cytokines secretion from macrophages and neutrophils
Kidney disease	Chronic kidney diseases Nephrolithiasis Acute kidney injury	Renal vasoconstriction via inflammation Oxidative stress and endothelial dysfunction Renin-angiotensin system activation by UA
Metabolic syndrome (MetS)	Central obesity Hypertension Hyperglycemia Low HDL cholesterol	Insulin resistance and dyslipidemia Excessive consumption of uric acid Renal dysfunction Oxidative stress and inflammation
Cardiovascular disease (CVD)	Hypertension Atrial fibrillation Coronary heart disease Herat failure	Endothelial dysfunction and chronic inflammation XO effects ischemic and other types of tissue Vascular injuries Inflammatory diseases
Hypertension	High blood pressure	Oxidative stress Intracellular urate activity Endothelial dysfunction and vascular damage
Intervertebral degeneration (IVD)	Back pain and stiffness Nerve compression and numb Limitation of range motion	Oxidative stress and inflammation Microvascular dysfunction Cellular damage
Diabetes	Obesity Insulin resistance Peripheral neuropathy	Insulin resistance and hyperinsulinemia High consumption of Fructose Oxidative stress and inflammation

### Mendelian randomized studies

A significant biomedical question is whether hyperuricemia is causally associated with related comorbid conditions such as gout, hypertension, cardiac and kidney disease, etc.^202–209^ Utilizing data from observational epidemiologic studies in conjunction with experimental evidence from in vitro and animal model investigations, elevated serum urate levels have been suggested as being potentially linked to concurrent metabolic disorders.^210^ The principles of Mendelian randomization, which leverages genetic variants influencing exposures (e.g., UA levels), can serve as a natural randomization method to study the causal effects of these exposures on disease outcomes.^21,211,212^ To investigate the association between elevated serum urate concentrations and comorbid metabolic conditions, Mendelian randomized studies were conducted using genetic variants linked to increased serum urate levels.^213–215^ These genetic variants act as proxies for prolonged urate exposure, assuming they remain unconfounded by other factors.^18,22,206,216^ The pioneering Mendelian randomization studies leveraged specific genetic variants with substantial impacts on serum uric acid levels as instrumental variables. The objective of their research was to investigate the associations between uric acid concentrations and various health conditions, including body mass index, bone mineral density, coronary artery disease, blood pressure, metabolic syndrome, blood glucose levels, triglyceride levels, diabetes mellitus, serum creatinine levels, estimated glomerular filtration rate, Parkinson’s disease, memory, and gout.^207,212,217–220^ However, over the past three years, Mendelian randomization studies have utilized genetic variants associated with serum uric acid levels, identified through genome-wide association studies (GWAS), to construct genetic risk scores. These investigations consistently indicate a lack of evidence supporting a causal relationship between elevated serum urate levels and the risk of developing type 2 diabetes mellitus, coronary heart disease, ischemic stroke, and heart failure.^17,221–223^ Li et al.^22,169^ conducted a comprehensive analysis of 107 Mendelian randomization studies, included a median of 7,158 participants and 2,225 cases, with serum uric acid level as the exposure variable for various health outcomes. The instrumental variables utilized in these studies explained 2% to 6% of the variability in serum uric acid levels. The results indicated a significant association between serum uric acid levels and four health outcomes: diabetic macrovascular disease, arterial stiffness, renal events, and gout. Particularly noteworthy was the robust association observed with gout. However, the study did not find significant associations with several common cardiac and metabolic disorders, including type 2 diabetes, hypertension, chronic kidney disease, ischemic heart disease, and congestive heart failure.^224–238^ These findings suggest that while elevated serum uric acid levels may be associated with certain health outcomes such as gout and renal diseases, the evidence does not strongly support a causal relationship with other metabolic disorders. Additional analyses have shown consistent results across most outcomes examined, which included a variety of cardiovascular diseases, such as incidence of atrial fibrillation,^239^ coronary heart disease, incidence of hypertension,^216^ and incidence of stroke,^71^ diabetes,^240^ chronic kidney disease,^222^ mild cognitive impairment, Parkinson’s disease,^241^ and multiple sclerosis.^21^ However, statistical significance was inconsistent in the two outcomes of diabetic neuropathy^5,218^ and Alzheimer’s disease.^242^ In particular, the role of genetic variants, such as those within the SLC2A9 gene, in influencing cardiovascular and metabolic outcomes remains subject to debate.^6,191,223,243,244^ Recent research has delved into the causal relationship between variants of the URAT1 transporter gene (SLC22A12) and obesity and metabolic syndrome.^245,246^ In a randomized controlled trial involving patients with essential hypertension, specific SLC22A12 single nucleotide polymorphisms (SNPs), such as rs11602903, were associated with higher body mass index (BMI), larger waist circumference, higher HDL cholesterol levels, and the presence of metabolic syndrome in individuals of European descent.^247–249^ However, these associations were not observed in non-European populations, underscoring potential ethnic differences in genetic susceptibility to hyperuricemia-related metabolic abnormalities.^250–252^

### UA induced inflammation and relative mechanism

**Fig. 5:**
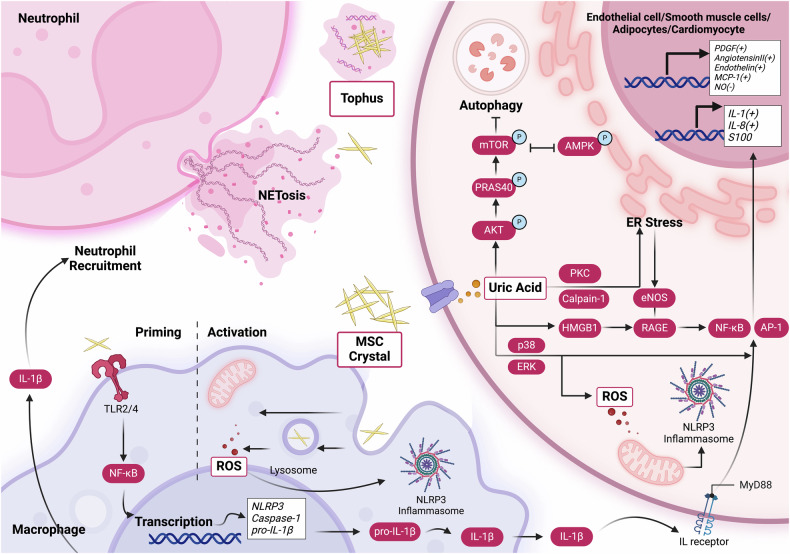
The role of uric acid in the pathogenesis of hyperuricemia and its associated diseases involves complex intracellular signaling mechanisms. Elevated intracellular uric acid levels stimulate the production of reactive oxygen species and activate multiple inflammatory signaling pathways. XO xanthine oxidase, eNOS endothelial nitric oxide synthase, MSU monosodium urate, Nrf2 Nuclear factor-erythroid 2-related factor 2, mTOR mammalian target of rapamycin complex, ERK extracellular signal-regulated kinase, AMPK AMP-activated protein kinase, IL-1β interleukin-1β, MAPK mitogen-activated protein kinases, PRAS40 Proline-Rich AKT Substrate, NF-κB nuclear factor κB, TLR Toll-like receptors, NLRP3, NOD-, LRR- and pyrin domain-containing 3, PKC Protein Kinase C, RAGE Receptor for Advanced Glycation End Products pathway

Uric acid signaling triggers the activation of several transcription factors, such as NF-κB or AP-1, through the activation of MAPK p38 and ERK pathways, resulting in the production of reactive oxygen species (ROS) under the conditions of hyperuricemia.^78^ The NLRP3 inflammasome, part of the nucleotide-binding domain and leucine-rich repeat protein family complex, is essential in the development of numerous infections and inflammatory disorders.^116,253–256^ The expression of NLRP3 is induced by NF-κB activation, leading to the assembly of a complex with the adaptor protein ASC and procaspase-1.^85,91,114,257^ Subsequently, procaspase-1 transforms into its mature form, caspase-1. This enzyme then activates pro-IL-1β and pro-IL-18, converting them into their mature forms, IL-1β and IL-18, respectively. This process coincides with the initiation of pyroptosis, facilitating the release of IL-1β into the extracellular environment.^258,259^ In gout, the activation of the NLRP3 inflammasome by monosodium urate (MSU) crystals stimulates the release of IL-1β, which contributes to the progression of arthritis. This activation mechanism involves phagocytic cells such as macrophages and neutrophils.^260–264^ This cascade of events leads to enhanced transcription of innate cytokines in various cell types including vascular endothelial cells, smooth muscle cells, and adipocytes. The activation is linked to the generation of vasoconstrictive agents, including MCP-1, (pro)renin receptor, endothelin, and angiotensin II, while concomitantly diminishing vasodilatory compounds like nitric oxide, which may contribute to the development of hypertension and lead to decreased viability of cardiomyocytes and myocardial damage.^94,265–267^ Moreover, in vascular cells, upregulation of growth factors like PDGF has been noted, which can promote smooth muscle cell proliferation and atherosclerosis.^268^ In vascular smooth muscle cells, uric acid-induced activation of MAPKs promotes the expression of MCP-1, an important chemokine involved in atherosclerosis progression. In pancreatic β-cells, uric acid triggers ERK activation, resulting in reduced cell viability, apoptosis, and the production of reactive oxygen species.^78,269,270^ Treatment with a URAT1 inhibitor suppresses the ERK pathway and mitigates uric acid-induced cell damage, underscoring the involvement of intracellular uric acid in MAPK activity.^271–274^ Additionally, uric acid regulates MAPK through phosphatase activity that inhibits the MAPK pathway.^275,276^ Monosodium urate crystals are ingested by monocytes via phagocytosis, engaging Toll-like receptors (TLRs) such as TLR2 and TLR4. This interaction prompts the recruitment of the adaptor protein ASC to the NLRP3 inflammasome complex. Subsequently, caspase-1 is drawn to the ASC assembly, where it oligomerizes along the ASC filaments. This oligomerization triggers the autoproteolytic maturation of caspase-1, activating its inflammatory caspase function.^183,277^ Active caspase-1 then catalyzes the proteolytic cleavage and maturation of proIL-1β into the biologically active IL-1β, which leads to acute flares of gouty arthritis.^183^ In neutrophils, uric acid activates the ERK/p38 signaling pathway while inhibiting the Nrf2 pathway. Additionally, monosodium urate crystals induce the translocation of Nrf2 into the nucleus and modulate intracellular reactive oxygen species levels, thereby promoting the activation of the NLRP3 inflammasome. This ROS-induced injury can lead to apoptosis, disruptions in ion regulation, and mitochondrial dysfunction, further exacerbating the inflammatory response and tissue damage.^67,278^ Furthermore, resolution of gout flares involves the formation of neutrophil extracellular traps, which capture monosodium urate crystals. Uric acid can modulate cytokine production and inflammatory outcomes through various pathways.^253,279,280^ Uric acid can enhance AKT phosphorylation, which subsequently leads to PRAS40 phosphorylation with the activation of mTOR,^281^ resulting in the inhibition of autophagy, as well as inhibiting AMPK phosphorylation.^282^ When UA levels are elevated, RAGE signaling is stimulated, leading to the activation of nuclear factor-kappa B (NF-κB). NF-κB activation triggers the transcription and release of pro-inflammatory cytokines within endothelial cells. Additionally, UA-induced activation of RAGE promotes the expression and extracellular release of high mobility group box 1 protein (HMGB1) by endothelial cells, lymphocytes, monocyte-derived macrophages, and vascular smooth muscle cells.^87,283,284^ The interaction between HMGB1 and RAGE amplifies the inflammatory cascade, contributing to cell apoptosis and endothelial dysfunction, resulted to CVD or CKD.^277,285^ This dysregulation of the HMGB1-RAGE pathway further diminishes NO availability, exacerbating inflammation. Moreover, UA-induced inflammation and oxidative stress can also trigger endoplasmic reticulum (ER) stress, decreased nitric oxide bioavailability and produce peroxynitrite (ONOO-), a very powerful radical, which contributing to cellular dysfunction and apoptosis.^61,103,270,282^ Uric acid diminishes nitric oxide levels through several mechanisms, including the consumption of NO due to excessive reactive oxygen species production and direct inhibition of NO synthesis. UA-induced dephosphorylation of endothelial NO synthase (eNOS) via uric acid transporters reduces NO production in human umbilical vein endothelial cells. Moreover, the HMGB1-receptor for advanced glycation end products pathway regulates eNOS production^14,98,286,287^ (Fig. 5).

### Biomarkers of hyperurceimia and its relative disease

Recent advances in proteomics have shed light on the biochemical underpinnings of hyperuricemia. Notably, Liu’s^288^ research indicated elevated serum levels of complement C3, haptoglobin, complement C4, and apolipoprotein A1 (apo A1) in Uyghur patients with hyperuricemia. This suggests a correlation between hyperuricemia and high-density lipoprotein (HDL) components, with apo A-I implicated in cholesterol transport and anti-atherosclerotic properties.^289–291^ Furthermore, HDL’s role in lipid metabolism regulation and its influence on cardiovascular disease and diabetes development cannot be overlooked.^292^ The inhibition of apo A-I may be linked to atherosclerosis progression through chronic inflammation pathways. Moreover, the complement system’s activation in response to hyperuricemia has been implicated in the pathogenesis of various inflammatory conditions, including gout, renal injury, and type 2 diabetes (T2DM).^144^ The NLRP3 inflammasome’s activation and its interplay with the complement and coagulation systems are of particular interest. Component C5a of the complement system, recognized for its potent pro-inflammatory effects, can amplify monocyte and neutrophil activation. MSU crystals have been shown to trigger IL-1β production and inflammatory cytokine release through C5a activation, highlighting the potential of complement antagonists in managing gout inflammation. In the realm of urine proteomics, Huo et al.^293^ conducted a comparative study between healthy individuals and those with hyperuricemia, revealing differentially expressed proteins that hint at pathways involved in insulin receptor recycling and lipid metabolism. The study pinpointed the V-type proton ATPase subunit B kidney isoform and Complex Factor D (CFAD or adipsin) as key factors impacting insulin regulation in hyperuricemia patients.

### Cardiovascular metabolic mechanisms and diseases

In recent years, numerous studies have reinforced the strong association between hyperuricemia and cardiovascular events. Research has demonstrated that serum uric acid levels are positively correlated with hypertension.^265,294,295^ The proposed mechanisms involve the activation of the renin-angiotensin system, inflammatory responses, oxidative stress, vascular smooth muscle cell proliferation, and insulin resistance.^57,69,78,94,296^ The ONATA study showed a negative correlation between serum uric acid levels and insulin sensitivity, suggesting a potential link between uric acid, insulin sensitivity, and the risk of developing hypertension.^105,212^ Hyperuricemia frequently coexists with insulin resistance, which can elevate the activity of the renin-angiotensin-aldosterone system and the sympathetic nervous system. This interaction leads to sodium retention, increased blood volume, and subsequent hypertension.^297,298^ In patients with hyperuricemia, elevated serum levels of high-sensitivity C-reactive protein (hs-CRP) are closely linked to inflammation and oxidative stress, which may exacerbate hypertension.^299^ Soluble urate may further exacerbate vascular inflammation and oxidative stress by promoting LDL oxidation, lipid peroxidation, and elevating hs-CRP levels. Moreover, hs-CRP can contribute to vascular endothelial injury by activating the complement system.^99,287^ Furthermore, elevated uric acid levels have been implicated in causing inflammation in endothelial cells via the activation of the receptor for advanced glycation end products (RAGE) signaling pathway.^285^ When UA levels are increased, RAGE signaling becomes activated, that ultimately lead to endothelial dysfunction, is a key feature of various cardiovascular diseases. Specifically, the activation of RAGE triggers the nuclear factor-kappa B pathway, resulting in the transcription and release of proinflammatory cytokines within endothelial cells.^277,285^ To mitigate UA-induced endothelial dysfunction and inflammation, targeting the RAGE signaling pathway offers a promising therapeutic strategy. Employing anti-RAGE antibodies to inhibit RAGE activity can suppress the HMGB1/RAGE signaling axis, thereby alleviating endothelial dysfunction and diminishing inflammation within endothelial cells.^285,300,301^(Fig. 5) By modulating this pathway, it may be possible to alleviate the detrimental effects of elevated UA levels on endothelial function and reduce associated cardiovascular risks.^105,265,302^

Elevated serum uric acid levels heighten the risk of cardiovascular disease (CVD) mortality, potentially due to mechanisms by which hyperuricemia activates the renin-angiotensin system and induces hypertension. Additionally, uric acid has been detected in atherosclerotic plaques.^102,201^ A recent study conducted in the Japanese revealed that even among healthy, lean, normotensive individuals, the presence of hyperuricemia is associated with an elevated risk of cardiometabolic disease.^191^ Kleber et al. reported a significant association between each 1 mg/dL increase in genetically predicted uric acid concentration and the risk of cardiovascular death and sudden cardiac death.^207^ Emerging evidence has linked elevated serum uric acid levels to cardiovascular diseases, particularly atherosclerosis and hypertension. Chronic low-grade inflammation is a hallmark of atherosclerosis, and UA-induced IL-1β release may contribute to this inflammatory milieu.^69,90,239,303^ Additionally, uric acid has been associated with endothelial dysfunction, which further exacerbates vascular inflammation and contributes to hypertension.^237,304–308^ Endothelial dysfunction characterized by impaired nitrogen oxide-mediated vasodilatation is a key event in the development of atherosclerosis.^94^ Experimental and clinical research has substantiated that elevated uric acid levels exert detrimental effects on cardiovascular health, with increased oxidative stress being a key mechanism implicated in these adverse outcomes,^6,66^ decreased nitric oxide availability, endothelial dysfunction, the promotion of local and systemic inflammation, vasoconstriction, vascular smooth muscle cell proliferation, insulin resistance^309^ and metabolic disorder.^51,124^ Additionally, endothelial dysfunction associated with elevated serum uric acid levels leads to a low-grade inflammatory state and vascular activation of the angiotensin system. As estrogen production declines with age in females, its cardio-protective effects may diminish, thereby increasing susceptibility to elevated uric acid levels.^307^

### Renal metabolic mechanisms and diseases

Emerging evidence increasingly supports the pathogenic role of hyperuricemia in both the onset and progression of chronic kidney disease (CKD). Untreated hyperuricemia is notably acknowledged as a risk factor for the development of CKD. In China, the prevalence of hyperuricemia among CKD patients varies from 36.6% to 50%, with a notable rise observed as CKD progresses.^310^ The mechanisms by which hyperuricemia contributes to chronic kidney disease include renal inflammation, endothelial dysfunction, and activation of the renin-angiotensin system.^13^ Hyperuricemia is known to stimulate the renin-angiotensin system and impair endothelial nitric oxide release, which collectively lead to renal vasoconstriction and increased blood pressure.^100^ Nitric oxide (NO) plays a crucial role in regulating vascular endothelial cell relaxation, maintaining stable renal vascular tone, and influencing renal blood flow, renin secretion, and tubuloglomerular feedback mechanisms.^105,311^ However, hyperuricemia inhibits nitric oxide synthase, leading to reduced nitric oxide levels.^312^ Furthermore, endothelial cells respond to hyperuricemia by upregulating angiotensin-converting enzyme activity, which enhances angiotensin II and superoxide anion production. This cascade promotes vasoconstriction and hypertension.^102,313^ Uric acid directly affects endothelial cells by reducing nitric oxide levels, influencing processes such as vascular smooth muscle cell proliferation, extracellular matrix deposition, and the adhesion and migration of macrophages.^102,227,228,314–317^ These effects lead to arterial resistance and remodeling, ultimately contributing to renal dysfunction and fibrosis.^6,318^ However, the evidence supporting the treatment of asymptomatic hyperuricemia in hypertensive patients with chronic kidney disease is limited. Observational studies have produced inconsistent findings, and there is a notable absence of large-scale randomized controlled trials to validate the efficacy of lowering uric acid levels. Despite these limitations, the majority of existing studies suggest that therapies aimed at reducing uric acid levels may potentially attenuate the progression of CKD.^102,319–321^ A single-center double-blind, randomized, parallel placebo-controlled study found that uric acid reduction slowed the decline of glomerular filtration rate in patients with stage 3 and 4 CKD.^322^ Another study by Jeong et al. demonstrated that febuxostat treatment to reduce serum uric acid levels tended to reduce renal functional deterioration in patients with both CKD and hyperuricemia.^323^ These findings suggest that reducing uric acid levels could potentially improve renal function. However, ongoing debate centers on whether the benefits of uric acid-lowering therapy stem from decreased uric acid levels or the inhibition of XO activity.^319,324,325^ Further investigations have shown that medications like benzbromarone and febuxostat can mitigate the advancement of chronic kidney disease and decrease serum uric acid levels in CKD patients, highlighting the potential advantages of treatments aimed at lowering uric acid levels.^319,326–328^

Increased uric acid levels are linked to kidney inflammation and the progression of kidney diseases, especially in the presence of hyperuricemia. Gout has been identified as an independent risk factor for chronic kidney disease, nephrolithiasis and acute kidney injury, wherein uric acid excretion by the kidneys participate in facilitating crystal-induced direct tubular toxicity.^310^ This finding underscores the close interconnection between uric acid and nitric oxide regulation in this particular clinical context.^329^ Kidney damage mediated by UA involves the stimulation of the renin-angiotensin-aldosterone system. In the medulla, an elevated UA concentration results in the deposition of urate precipitates and the activation of the Nod-like receptor protein 3 (NLRP3) inflammasome.^96^ Activation of these pathways leads to chronic interstitial inflammation and tubular damage, ultimately contributing to kidney fibrosis. Additionally, in the renal cortex, hyperuricemia amplifies the activity of the renin-angiotensin-aldosterone (RAA) system, fostering sustained vasoconstriction of the afferent arterioles.^130^ In turn, leads to glomerular damage and the development of glomerulosclerosis.^330^ CKD is characterized by endothelial dysfunction and NO deficiency; thus, uric acid is a potential contributor to CKD progression. It is primarily driven by the development of hypertrophy in the afferent arteriole, which compromises autoregulation and facilitates heightened transmission of systemic blood pressure to the glomerulus.^310^ The kidney’s susceptibility to oxidative stress stems from various sources including the mitochondrial respiratory complex, NADPH oxidases, endothelial nitric oxide synthase (eNOS), myeloperoxidase, and xanthine oxidoreductase (XOR), all of which contribute to the advancement of chronic kidney disease and its related complications.^331^ Oxidative stress is a characteristic feature of chronic kidney disease, initiating inflammation and endothelial dysfunction, which accelerates arteriosclerosis. This sequence plays a role in glomerular injury, leading to albuminuria and eventual glomerulosclerosis. Hyperuricemia exacerbates oxidative stress, thereby intensifying inflammation and endothelial dysfunction within this context.^332^

### Gout and its mechanism

Gout is one of the most common forms of chronic degenerative disease of the joints.^333,334^ It is defined by recurring episodes of inflammatory arthritis affecting joints and specific soft tissues, including cartilage, synovial bursae, and tendons, particularly in the lower extremities, due to the deposition of uric acid in the form of monosodium urate crystals.^34^ The painful pathological state of gout is mainly induced by hyperuricemia with the concentration of more than 6.8 mg/dL under physiological conditions (37 ◦C, pH 7.4).^333,334^ When uric acid levels increase to such concentration, crystals form in the joints, triggering an inflammatory response. MSU crystals display a triclinic structure composed of stacked sheets of purine rings, forming needle-shaped crystals observable under microscopy. The exposed, charged surfaces of these crystals are thought to promote interactions with phospholipid membranes and serum factors, thereby contributing to the inflammatory response mediated by these crystals.^335^ MSUs serve as the primary stimuli for initiating, amplifying, and sustaining the innate immune response. They are phagocytosed by macrophages as foreign particles and recognized by Toll-like receptors 2 and 4 (TLR2/TLR4), which subsequently activate and oligomerize the NLRP3 complex.^336^ The NLRP3 complex, a multiprotein assembly with proteolytic activity, facilitates the conversion of the pre-IL-1β into its active form, IL-1β. Subsequently, IL-1β is released into the extracellular milieu, initiating acute inflammation.^337^ The MSU crystals are initially engulfed by macrophages, which then facilitate the assembly and activation of the NLRP3 inflammasome, preceded by priming through pathways that activate NF-κB, such as those initiated by the engagement of Toll-like receptors (TLRs) within the TLR family.^338–340^ Inflammasomes are intracellular multiprotein complexes that trigger inflammatory responses. These structures emerge as intracellular pattern recognition receptors (PRRs) like NLRP3 detect signals that have infiltrated the cell’s cytosol.^341^ The recognition triggers the PRR to oligomerize and associate with a complex comprising adaptor proteins and effector enzymes.^342–344^ The formation of the NLRP3 inflammasome involves the recruitment of the adaptor protein ASC, which is then followed by the recruitment of caspase-1.^345^ Following initial oligomerization within the inflammasome structure, ASC monomers can subsequently polymerize into high-molecular-weight aggregates.^341,346^ The recruitment and oligomerization of caspase-1 by this complex initiate the activation and proteolytic cleavage of its substrates. Caspase-1 activates proinflammatory cytokines such as IL-1β by cleaving their respective precursor proteins and proIL-1β.^262^ In gout, the release of IL-1β mediated by inflammasomes triggers a significant inflammatory response characterized by vasodilation and rapid recruitment of neutrophils to the site of crystal deposition, thereby driving acute inflammatory episodes.^347–349^ Similarly, MSU promotes the expression of other cytokines, such as interleukin-6 (IL-6), tumor necrosis factor-α (TNF-α), and interferon gamma (IFN-γ), and chemokines, such as monocyte chemotactic protein-1 (MCP-1). MCP-1 induces the recruitment of innate immune cells and indirectly affects gout progression.^214^ Flare resolution involves the capture of MSU crystals by neutrophil extracellular traps.^350^

### Diabete and its mechanism

Chronic hyperuricemia is associated with pancreatic β-cell dysfunction, which is a critical component of type 2 diabetes.^4^ Research indicates that the likelihood of developing diabetes rises by 6% with each 1 mg/dL increment in uric acid concentration.^240^ Uric acid has been observed to adversely affect β-cells, resulting in impaired insulin secretion and decreased functional β-cell mass. This contributes to an insufficient compensatory response to insulin resistance and gluconeogenesis, mediated by the inhibition of hepatic AMP-activated protein kinase, thereby promoting the progression towards overt diabetes.^351^ Uric acid can promote oxidative stress, generating reactive oxygen species that cause cellular damage.^122^ Oxidative stress is closely associated with insulin resistance and β-cell dysfunction. The increased oxidative load in hyperuricemia may impair insulin sensitivity and exacerbate the metabolic disorders observed in diabetes.^309^ Moreover, hyperuricemia frequently coincides with low-grade inflammation. Increased uric acid concentrations have been implicated in the release of inflammatory cytokines, thereby contributing to systemic inflammation. Inflammation is a well-recognized contributor to the pathogenesis of insulin resistance and type 2 diabetes. This inflammatory environment disrupts insulin signaling pathways and exacerbates glucose intolerance. Elevated insulin levels, stemming from insulin resistance and β-cell dysfunction, may enhance the renal reabsorption of uric acid. Consequently, this cycle potentially reinforces higher serum uric acid levels, establishing a feedback mechanism in the interplay between hyperinsulinemia and hyperuricemia.^352^ Additionally, research has shown that elevated uric acid levels inhibit insulin-induced glucose uptake in cardiomyocytes. This effect is primarily mediated by an increase in the phosphorylation of insulin receptor substrate 1 (IRS1) and a concomitant inhibition of Akt phosphorylation, a crucial component of the insulin signaling pathway.^353^ Moreover, clinical research has identified a correlation between hyperuricemia and diabetes, although the causal relationship remains controversial. While high UA may accelerate the development of diabetes and impair glucose tolerance, it is insufficient to solely induce diabetes.^19,351,354^

### Metabolic syndrome (MetS) and its mechanism

Metabolic syndrome is characterized by a cluster of conditions, including obesity, insulin resistance, and dyslipidemia, and is intimately linked to chronic inflammation.^355^ The prevalence of metabolic syndrome rises by approximately 5% in men and 9% in female with each 1 mg/dL increase in serum uric acid concentration.^356^ Elevated serum uric acid levels have been shown to impair insulin sensitivity, thereby contributing to the development of insulin resistance. In response to increased insulin resistance, the pancreas compensates by secreting higher levels of insulin, resulting in hyperinsulinemia.^357^ The prevalence of metabolic syndrome components, including hyperglycemia, hypertriglyceridemia, low HDL cholesterol, and hypertension, shows a rising trend with increasing serum uric acid levels. Interestingly, central obesity appeared to decrease slightly in individuals with exceptionally high serum uric acid concentrations.^200^ Uric acid is implicated in the pathogenesis of metabolic syndrome through its ability to induce insulin resistance and promote low-grade inflammation, underscoring its proinflammatory role.^51^ In addition, hyperuricemia is frequently accompanied by dyslipidemia, another hallmark of metabolic syndrome. Studies have demonstrated an association between elevated serum uric acid levels and alterations in lipid profiles, including increased triglycerides and decreased high-density lipoprotein cholesterol (HDL-C) levels.^358^ These lipid abnormalities contribute to the dyslipidemia pattern often observed in individuals with metabolic syndrome. There is evidence to suggest that hyperuricemia may promote weight gain and central obesity, both of which are integral components of metabolic syndrome. Uric acid has been shown to influence adipogenesis and fat accumulation, potentially exacerbating obesity in individuals with MetS.^200^

### Hypertension and its mechanism

Hyperuricemia may acutely influence blood pressure through a renin-dependent pathophysiological mechanism. Additionally, it is postulated that hyperuricemia exacerbates hypertension by promoting systemic endothelial dysfunction and oxidative stress.^201^ Epidemiological studies consistently demonstrate a robust association between hyperuricemia and the incidence of hypertension. Each 1 mg/dL rise in serum uric acid is linked to a 13–15% increase in hypertension risk.^299^ Elevated serum uric acid levels constitute a substantial risk factor for both the onset and progression of hypertension. The precise mechanisms by which elevated uric acid concentrations lead to hypertension are not entirely understood but likely involve complex processes related to cardiovascular disease pathogenesis. The increase in blood pressure in hyperuricemic individuals is predominantly attributed to oxidative stress and intracellular urate activity, mediated chiefly by xanthine oxidoreductase (XOR).^299^ Uric acid deposition-induced inflammation, resulting in endothelial dysfunction and vascular damage (referred to as vascular gout), can occur at serum uric acid levels exceeding 6.5 mg/dL. This threshold is notably higher than those typically linked with hypertension and cardiovascular disease.^201^ Uric acid-lowering therapy effectively reduces both systolic and diastolic blood pressure in pediatric and adolescent patients newly diagnosed with essential hypertension, particularly in young individuals with a short duration of hypertension and preserved renal function.^200^

### Intervertebral degeneration (IVD) and its mechanism

Several studies have confirmed that the oxidative properties of physiological levels of UA can eliminate 60% of ROS in the body.^359^ This reduction in ROS can inhibit autophagy in IVDs and reduce the apoptosis of myeloid cells caused by oxidative stress, maintaining the stability of the structure of IVD.^94^ However, high concentrations of UA can promote oxidative stress and mitochondrial dysfunction. The induction of XO can promote the production of ROS, thus promoting oxidative stress in IVD and exacerbating IDD. Moreover, high osmolality induced by high uric acid levels may inhibit PDGF- and IGF-I-mediated DNA synthesis in the medulla oblongata.^268^ Additionally, MSU crystals may accumulate in IVD under high uric acid conditions and cause damage to cone endplates.^360^ MSU crystals cause cellular damage and mediate inflammatory responses, such as prostaglandin, bradykinin, IL-1, IL-6, and TNF-a.^337^ The accumulation of inflammation and bone destruction affect the ability of cartilage endplates to provide nutrients to IVD, exacerbating IDD.^361^

## Therapy approaches for hyperuricemia

Hyperuricemia can manifest either asymptomatically or symptomatically, leading to distinct management approaches.^95^ Thus, the management strategies for hyperuricemia typically involve two primary modalities: non-pharmacological interventions and pharmacological therapy. These approaches are tailored based on the clinical presentation and individual patient factors, aiming to mitigate the risk of complications.^362^

### Treatment of asymptomatic hyperuricemia

Non-pharmacological interventions play a crucial role in managing hyperuricemia, particularly in asymptomatic individuals.^363,364^ Dietary modifications, such as adherence to a low-purine diet and avoidance of alcohol, particularly beer and spirits, sugar-sweetened beverages, heavy meals, and excessive intake of meat and seafood, have demonstrated efficacy in reducing uric acid levels by approximately 10-15%.^365^ Furthermore, incorporating cherries, coffee, and low-fat dairy products into the diet can confer beneficial effects. Consumption of fructose-rich beverages should be minimized. Protein-rich vegetables like nuts, legumes, beans, spinach, cauliflower, and mushrooms can be consumed in moderation due to their low bioavailability of urate and high fiber content, which are less likely to elevate serum uric acid levels.^324,366–369^ However, complete prohibition of purine intake is not recommended due to its limited effect on serum uric acid levels (approximately 1 mg/dL) and the significant burden it imposes on patients.^1^ Instead, supplementation with vitamins such as ascorbic acid (vitamin C) and folic acid can help lower serum urate concentrations. Vitamin C, administered in doses ranging from 500 to 1000 mg/day, exhibits mild uricosuric properties and can complement dietary and lifestyle modifications. Similarly, folic acid supplementation has been shown to effectively reduce serum urate levels.^9,127,370,371^ Of note, non-pharmacological interventions represent valuable adjunctive measures for all individuals with gout, encompassing weight management and avoidance of excessive consumption of purine-rich foods, alcoholic beverages, and fructose-rich beverages. However, complete elimination of purine intake is not recommended due to its limited impact on serum uric acid levels, typically resulting in a reduction of approximately 1 mg/dL. Nonetheless, exceptional cases may require pharmacotherapy even in asymptomatic individuals with elevated serum uric acid levels.^372^ For instance, patients undergoing radiotherapy or chemotherapy for malignancies are at risk of uric acid nephropathy and may require preventive therapy with intravenous hydration and xanthine oxidase inhibitors.^330,373,374^ Xanthine oxidase inhibitors are typically used in such case. In Japan, treatment of asymptomatic hyperuricemia is recommended to mitigate the risk of chronic diseases such as hypertension, coronary artery disease (CAD), and chronic kidney disease (CKD).^191^ However, the decision to initiate pharmacotherapy for asymptomatic hyperuricemia remains debatable and should be based on individual risk factors and considerations. While recent studies suggest a potential association among hyperuricemia, cardiovascular and renal diseases, further research is needed to elucidate the mechanisms and clinical benefits of urate-lowering therapy in these populations.^102,320,375^

### Treatment of hyperuricemia with commodities

An analysis of twenty-two guidance documents revealed a consensus on target serum uric acid levels for long-term control, with 6.0 mg/dL (or 360 μmol/L) as the predominant recommendation.^362^ Uric acid-lowering drugs can be broadly classified into three major groups: drugs that reduce uric acid synthesis (xanthine oxidase inhibitors), drugs that promote uric acid excretion (reabsorption inhibitors), and drugs that regulate uric acid metabolic hydrolysis (uricase inhibitors).^1^ Irrespective of the specific urate-lowering therapy (ULT) selected, fundamental principles entail commencing treatment concurrently with prophylaxis and initiating with a conservative dosage, followed by systematic monitoring of serum uric acid levels and subsequent dose adjustment until the therapeutic target is attained.^310^ For symptomatic hyperuricemia, the common pharmacological interventions for urate-lowering therapy (ULT) are illustrated in Fig. 4. Xanthine oxidase (XO) plays a central role in purine metabolism by catalyzing the conversion of hypoxanthine to xanthine and further to uric acid (UA). Concurrently, XO contributes to the production of reactive oxygen species (ROS).^372^ Allopurinol, categorized as a purine-like XO inhibitor, and febuxostat and topiroxostat, classified as non-purine XO inhibitors, constitute the primary pharmacological approach for ULT.^326,376,377^ By inhibiting XO activity, these agents demonstrate antioxidant properties by reducing ROS production associated with purine metabolism as well as remain the cornerstone of hyperuricemia treatment.^139^ Uricosuric agents represent a secondary or alternative therapeutic option, with recent guidelines advocating their use in combination with XO inhibitors when monotherapy proves ineffective.^378^ However, it is crucial to acknowledge that the predominant cause of hyperuricemia in most patients which is impaired renal clearance of uric acid. This impairment may be influenced by inherited renal transport factors or a reduced estimated glomerular filtration rate (eGFR).^102,189,320,330^ In patients with lower eGFR levels, uricosuric agents may not be as effective, necessitating the use of alternative agents with different mechanisms of action.^379–381^ Benzbromarone, another potent uricosuric drug, acts by inhibiting URAT1 and GLUT9.^328,382^ Emerging evidence linking hyperuricemia to cardiovascular and metabolic comorbidities has spurred the development of novel agents. Lesinurad and arhalofenate, inhibitors of URAT1 and OAT4, respectively, offer promising therapeutic avenues.^383,384^ Dotinurad, a selective urate reabsorption inhibitor available in Japan, inhibits URAT1 with high selectivity, demonstrating non-inferiority to febuxostat in lowering serum UA levels with no significant safety concerns^385^ (Table 4).

**Table 4:** Update on the therapies for the treatment of hyperuricemia

Therapy	Characteristics	The Indications & Mechanisms	Limitations & Adverse Effects	Dosage & Uses	Clinical Benefits
Allopurinol^400,457,458^	Xanthine oxidase inhibitor	First-line therapy with wide availability, attaining the targeted UA concentrations is not consistently realized, attributed to various factors such as insufficient SU monitoring, poor adherence to medication, and inadequate dosing.	Hypersensitivity syndrome: rash, eosinophilia, leukocytosis, fever, hepatitis and progressive kidney failure. Eosinophilia, hepatitis, and interstitial nephritis. Severe cutaneous adverse reactions (SCARs).	Mild: 50–100 mg/day PO initially; Increased weekly to 200–300 mg/day; Moderate to severe: 100 mg/day PO initially; Increased weekly to 400-600 mg/day; Maximum PO dosage: 600 mg/day.	For gout treatment and prevention. Reducing cardiovascular and renal outcomes in patients with asymptomatic hyperuricemia. Improve renal function in children.
Febuxostat^400,457,458^	Xanthine oxidase inhibitor	A recommended urate-lowering therapy, marginally reduced risk of heart failure exacerbation.	Muscle pain, stomach. Discomfort, skin rashes, diarrhea, and elevations in liver enzymes.	Initial dose: 20–40 mg PO qDay; Increase to 80 mg PO qDay after 2 wk if serum uric acid <6 mg/dL is not achieved.	More effective in reaching the target of serum uric acid under 6 mg/ dl compared to allopurinol. Potential nephroprotective and cardioprotective effects. Lower risk of primary composite event (cerebral, cardiovascular, and renal events and all deaths).
Topiroxostat^406,459,460^	Xanthine oxidase inhibitor/ABCG2 inhibitor	Lowering UA levels while maintaining renal function and exhibiting a positive impact on urinary albumin excretion.	Polyarthritis, nasopharyngitis, and increases the risk of liver damage.	Generally: 20 to 80 mg twice daily.	Reduce left ventricular end-diastolic pressure. Exert nephroprotective properties.
Probenecid^461,462^	URAT1 and OAT1, OAT3 inhibitor	For the treatment of renal impairment, it impedes the renal elimination of organic anions and concurrently disrupting tubular urate reabsorption.	Gastrointestinal upset, allergic reactions, nephrolithiasis, hypersensitivity reactions.	Moderate to severe: 250 mg PO twice daily for 1 week; Increase to 500 mg PO twice daily to 2 g/day maximum with dosage increases of 500 mg q4 weeks.	Improved cardiac function and increased in vitro cardiomyocyte calcium sensitivity.
Benzbromarone^382,414,463^	URAT1 and GLUT9 inhibitor	Lower development of CKD compared to allopurinol. Relevant for addressing renal dysfunction in hyperuricemia.	Rash, elevation in liver enzymes, hepatotoxicity.	Initial dose:12.5–50 mg daily; Maximum PO dosage: 100 mg/day.	Reduced risk of kidney disease progression and lower risk of end-stage renal disease. Reduction in the risk of developing the first gout flare and type 2 diabetes. Reduced endothelial dysfunction.
Lesinurad^464^	URAT1 and OAT4 inhibitor	Indicated in combination with a xanthine oxidase inhibitor for hyperuricemia associated with gout in patients who have not achieved target serum uric acid levels with a xanthine oxidase inhibitor alone.	Kidney failure, cardiovascular events.	Maximum dose: 200 mg PO qDay in combination with a xanthine oxidase inhibitor.	Considered as an add-on-therapy if the serum uric acid target is not reached with XO inhibitors.
Dotinurad^420^	URAT1 inhibitor	Maintaining a strong serum uric acid lowering effect with less safety concerns, compared to other agents like benzbromarone.	Gouty arthritis and bursitis. Tend to cause kidney damage.	Oral daily dose: 0.5 mg to 4 mg.	Safety in patients with an estimated glomerular filtration rate (eGFR).
Arhalofenate^419^	NSAID; URAT1 and OAT4 inhibitor;	The peroxisome proliferator-activated receptor ligand γ modulator that lowers IL-1β levels to offer an additional advantage by potentially decreasing and preventing gout.	Nephrolithiasis potential liver function abnormalities, cardiovascular risks.	Oral daily dose: 200, 400, or 600 mg once/daily.	Significantly reduce the number of gout flares.
Rasburicase^465^	Uricase	Receiving anticancer therapy expected to result in tumor lysis and subsequent elevation of plasma uric acid, not recommended for asymptomatic hyperuricemia.	Uncertain benefits in managing tumor lysis syndrome (TLS) in cancer patients. The adverse effect include anaphylaxis and methemoglobinemia.	Initial dose: 0.2 mg/kg IV infused over 30 min qDay for up to 5 days.	For the prevention and treatment of tumor lysis syndrome.
Pegloticases^430^	Uricase	Approved for use in adults with gout resistant to conventional therapy, where UA levels remain elevated despite maximum appropriate doses of xanthine oxidase inhibitors or when the use of xanthine oxidase inhibitors is contraindicated.	Infusion reactions, gout flares and anaphylaxis. Increased risk of cardiovascular events.	Initial dose: 8 mg IV infusion q2wk coadministered with methotrexate 15 mg PO qWeek.	In patients with refractory tophaceous gout.
Tranilast^422,424^	Anti-inflammatory agent; URAT1, GLUT9 inhibitor	Inhibiting renal transporters URAT1 and GLUT9.	Liver impairment, immune thrombocytopenia, eosinophilic cystitis, and eosinophilic polymyositis.	Varying doses (300 mg, 600 mg, and 900 mg/per day.	Reducing urate crystal associated inflammation.
Ulodesine (BCX4208)^427^	Inhibitor of purine nucleoside phosphorylase	Inhibiting PNP, ulodesine reduces the substrates available for XO to form uric acid.	Its potential impact on T cells.	Response rates for doses of 5, 10, and 20 mg were 40%, 50%, 45%, and 65%.	Synergistic action when combined with allopurinol, causes dose-dependent reduction in xanthine and hypoxanthine.

### Xanthine oxidase inhibitors (XOIs)

Xanthine oxidase catalyzes the conversion of purine metabolites to uric acid (UA). Therefore, xanthine oxidase inhibitors reduce UA production from both endogenous and dietary purine sources, making them the first-line therapies for managing hyperuricemia.^23,55^

#### Allopurinol

Allopurinol is an inhibitory agent that interferes with xanthine oxidase-mediated purine synthesis; allopurinol undergoes metabolic conversion to alloxanthine, which is a potent xanthine oxidase enzyme inhibitor.^386^ The dual action of allopurinol and its metabolite, alloxanthine, inhibits xanthine oxidase, effectively catalyzing the conversion of hypoxanthine to xanthine and its subsequent conversion to uric acid. Allopurinol is crucial for promoting the secondary utilization of hypoxanthine and xanthine through a metabolic pathway intricately linked with hypoxanthine-guanine phosphoribosyl transferase. This metabolic cascade contributes to the synthesis of nucleic acids and nucleotides, elucidating the multifaceted impact of allopurinol on purine metabolism.^358^ This metabolic process results in an increased concentration of nucleotides, triggering feedback mechanisms that suppress the synthesis of purines. Consequently, the reduced levels of uric acid in both urine and serum contribute to a reduction in the occurrence of hyperuricemia.^383^ The initial dose of 50 mg was given 1 ~ 2 times a day, and each time, the dose was increased by 50 ~ 100 mg. The general dose was 200 ~ 300 mg/d, divided into 2 ~ 3 doses, for a maximum daily dose of 600 mg.^387^ Notably, the CKD-FIX study and the PERL trials demonstrated that allopurinol did not significantly slow the deterioration of estimated glomerular filtration rate (eGFR) in chronic kidney disease patients compared to placebo, over a span of 2 and 3 years, respectively.^388,389^ However, a contrasting outcome was observed in a pediatric study where allopurinol treatment over four months led to an improvement in renal function in children with CKD.^390^ The ALL-HEART trial, which included patients over the age of 60 with ischemic heart disease but without a history of gout, found that allopurinol did not reduce the incidence of non-fatal myocardial infarction, non-fatal stroke, or cardiovascular death over an average follow-up period of 4.8 years when compared to standard care.^391^ Conversely, a smaller study involving 82 heart failure patients indicated that long-term allopurinol administration was associated with an improvement in left ventricular function.^392^ However, an algorithm was developed to theoretically mitigate the risk of allopurinol hypersensitivity syndrome (AHS), which occurs in 2–8% of patients.^393^ Allopurinol can cause severe cutaneous adverse reactions (SCARs), which is a primary concern associated with allopurinol use. SCARs are strongly correlated with the HLA B*5801 allele and include drug rash accompanied by eosinophilia and systemic symptoms, Stevens–Johnson syndrome, and toxic epidermal necrosis.^394^ In certain regions, a precautionary measure involves tailoring the allopurinol dosage based on creatinine clearance, aiming to mitigate the risk associated with SCARs, particularly considering renal failure as a predisposing factor for these adverse reactions. Notably, when allopurinol doses are less than 300 mg daily, less than half of patients achieve the therapeutic target of a serum uric acid (SU) concentration less than 6.0 mg/dL.^395^ Intriguingly, even in instances where allopurinol is not adjusted according to the estimated glomerular filtration rate (eGFR), approximately one-third of patients fail to attain SU levels below the designated threshold of 6.0 mg/dL. Nonetheless, a significant portion of patients receiving allopurinol fail to reach the target serum urate concentrations.^396^ Notably, the ABCG2 Arg141Lys variant has been consistently linked with poor response to allopurinol across multiple independent cohorts.^397^

#### Febuxostat

Febuxostat, a nonpurine xanthine oxidase inhibitor, effectively reduces serum uric acid levels without impeding the enzymes involved in pyrimidine and purine metabolism and synthesis. Its biotransformation is facilitated by uridine diphosphate glucuronosyltransferase (UGT) enzymes. Notably, patients treated with febuxostat demonstrate a slightly reduced risk of heart failure exacerbation compared to those receiving allopurinol.^398^ In China, febuxostat is recommended as a urate-lowering therapy and is prescribed at a daily dosage of 20–40 mg, contrasting with the global recommendation of 80-120 mg/day. In the United States, febuxostat is tolerated at doses of 40 and 80 mg per day, while in Europe it is 120 mg per day, and in Japan it is 10–60 mg per day.^336^ The CONFIRMS trial provided evidence that a daily dosage of 80 mg of febuxostat is more efficacious in lowering serum uric acid levels than a 300 mg daily dose of allopurinol.^399^ This nonpurine xanthine oxidase inhibitor is considered a viable alternative for individuals with allopurinol allergies. Notably, in scenarios of renal impairment, febuxostat is deemed more potent than allopurinol.^326^ Approximately 65% of patients achieved serum uric acid levels less than 6.0 mg/dL, although concerns have been raised regarding the cardiac safety profile of febuxostat.^400^ Common side effects of febuxostat encompass muscle pain, gastrointestinal discomfort, skin rashes, diarrhea, and a slight elevation in liver enzymes. Notably, the incidence of skin rashes is comparable to that observed with allopurinol. The manufacturer advises careful monitoring of liver function at the commencement of therapy and in the presence of any symptom’s indicative of liver injury. Importantly, the incidence of adverse effects associated with febuxostat remains low unless the daily dosage exceeds 120 mg.^376^ In the PRIZE trial, a cohort of 483 individuals with asymptomatic hyperuricemia was randomly assigned to either a two-year treatment with febuxostat or a control group that received lifestyle modifications. The study found that febuxostat did not correlate with a slowed progression of carotid intima-media thickness.^401^ However, a detailed sub-analysis revealed that the febuxostat group experienced a greater reduction in arterial stiffness parameters compared to the control group.^402^ Additionally, another sub-analysis indicated that patients on febuxostat treatment exhibited improved diastolic function.^403^ The FREED trial, involving 1070 elderly patients with hyperuricemia and cardiovascular risk factors, randomized participants to receive either febuxostat or non-febuxostat treatment. The results demonstrated that febuxostat was linked to a significantly reduced risk of the primary composite endpoint, encompassing cerebral, cardiovascular, renal events, and all-cause mortality.^404^

#### Topiroxostat

Topiroxostat, a nonpurine xanthine oxidase inhibitor, interacts with multiple amino acid residues within the solvent channel and forms covalent bonds with the molybdenum ion. This interaction produces a hydroxylated 2-pyridine metabolite that effectively inhibits xanthine oxidase, a critical enzyme in uric acid metabolism. Additionally, topiroxostat inhibits the ATP-binding cassette transporter G2 (ABCG2), which plays a key role in renal uric acid reabsorption and the secretion of uric acid from the intestines.^394^ However, topiroxostat has not received approval for use in the United States and Europe, although it is utilized in Japan. The medication is available in oral tablets of 20, 40, and 60 mg. The standard recommendation is to initiate treatment with a 20 mg dose administered twice daily, with a maximum approved dosage of 80 mg twice daily.^387^ The literature on topiroxostat reports certain adverse effects, including polyarthritis, nasopharyngitis, and an increase in liver enzymes. Notably, the majority of these adverse effects are generally classified as mild to moderate in severity.^377^ A study demonstrated a marked enhancement (≥150%) of warfarin activity in 32% of patients with cardiovascular disease and hyperuricemia who were treated with topiroxostat.^405^ A prospective, randomized, blinded study compared the effects of topiroxostat with allopurinol and suggested that topiroxostat may lead to a reduction in left ventricular end-diastolic pressure, indicating a potential benefit for cardiac function.^406^ Additionally, the study hinted at nephroprotective properties of topiroxostat compared to allopurinol. As for nephroprotection, an RCT on the use of febuxostat vs topiroxostat show none improvement in urinary protein/creatinine ratio.^407^

### Advanced therapy

Hyperuricemia can arise due to either overproduction or underexcretion of uric acid, with the latter being the predominant form as mentioned. The underexcretion of uric acid is primarily attributed to diminished renal clearance.^138,139^ During renal filtration, uric acid is extensively handled by proximal tubular cells. Approximately 90% of the filtered uric acid is reabsorbed via the apical transporters URAT1 and OAT4, as well as the basolateral GLUT9. Conversely, a portion of uric acid is secreted back into the proximal tubular lumen through various apical transporters, including ABCG2, NPT1, NPT4, and GLUT9, along with the basolateral transporters OAT1 and OAT3.^138,139^ Uricosuric agents act on the proximal tubule of the kidney by inhibiting the reabsorption of uric acid or enhancing its excretion. These medications are considered second-line treatments for hyperuricemia, particularly in cases unresponsive to standard therapies. They are often used in conjunction with xanthine oxidase (XO) inhibitors or prescribed for patients who cannot tolerate XO inhibitors. Additionally, certain antihypertensive and lipid-lowering drugs, such as losartan, simvastatin, atorvastatin, and fenofibrate, have been shown to reduce serum uric acid levels, potentially exhibiting a synergistic effect when used alongside standard hypouricemic treatments.^81,408^

#### Probenecid

Probenecid is a quintessential uricosuric agent with multifaceted effects on renal function, significantly influencing the elimination of organic anions and tubular reabsorption of urate. Its therapeutic potential extends beyond managing hyperuricemia, demonstrating efficacy as a URAT1 and GLUT9 inhibitor, especially in cases of renal impairment. Probenecid exerts its uricosuric effects by inhibiting renal organic anion elimination and disrupting tubular urate reabsorption. This dual action enhances urinary uric acid excretion, thereby reducing serum urate concentrations. Additionally, probenecid may modulate urate binding by plasma proteins and influence uric acid secretion within the renal tubules.^397^ The comprehensive use of probenecid is not without consideration of adverse reactions, as it spans various organ systems. Gastrointestinal, dermatologic, hematologic, renal, and immunologic manifestations have been reported.^409^ Approximately 5% of users experience manifestations such as rash, gastrointestinal complaints, and hypersensitivity reactions. While serious toxicity is infrequently reported, a notable proportion of patients, approximately one-third, may exhibit intolerance, necessitating discontinuation of probenecid.^410^

#### Benzbromarone

Benzbromarone, recognized as a uricosuric agent, exhibits notable in vitro inhibitory effects on urate transport facilitated by URAT1 and GLUT-9. Its approval for clinical use comes with cautious considerations, particularly in terms of dosing and associated hepatotoxicity. The recommended starting dosages of benzbromarone range from 12.5 mg to 50 mg daily, a regimen predominantly observed in Europe and Asia.^79,83,411^ As novel URAT1 inhibitor uricosuric therapies gain momentum in clinical trials, particularly among Western populations, the application of benzbromarone warrants meticulous consideration. Patient selection for clinical trials should reflect the pathophysiologic subtype of hyperuricemia, existing comorbidities, and concurrent use of urine alkalization agents such as potassium citrate. This comprehensive approach facilitates a thorough evaluation of benzbromarone’s efficacy and safety across diverse patient cohorts, ultimately refining uricosuric therapy regimens.^55^ The risk of hepatotoxicity is especially increased in individuals administered high doses of 300 mg daily of benzbromarone. However, findings from a comprehensive systematic review indicates that, when juxtaposed with probenecid, benzbromarone is associated with a reduced frequency of adverse effects.^378^ Certain scholars posit that the removal of benzbromarone from the market may not align with the best interests of patients with gout, with the argument that conceivable toxicity could be mitigated through a cautious approach involving gradual dosage escalation coupled with vigilant monitoring of liver function.^327^ Importantly, similar to those treated with febuxostat, patients on benzbromarone exhibited a significantly reduced risk of advancing to end-stage renal disease.^412^ In an additional study, benzbromarone demonstrated a lower risk of experiencing the initial gout flare and developing type 2 diabetes when contrasted with allopurinol.^413^ A randomized, open-label, crossover study comparing benzbromarone and febuxostat in hyperuricemia patients indicated that benzbromarone was associated with diminished endothelial dysfunction and an increase in adiponectin levels.^414^

### Emerging drugs and uricosuric compounds

Given the advancements in drug delivery systems and a deeper understanding of renal mechanisms and urate transporters, numerous novel therapeutic agents for managing hyperuricemia are currently in various stages of clinical development.^372,415,416^ Emerging pharmacological agents for hyperuricemia management are currently undergoing various stages of clinical development, ranging from preclinical to early clinical trials. Notable candidates in Phase II/III trials include arhalofenate (MBX102), AC201, the RDEA group (including lesinurad), tranilast, and ulodesine (BCX4208). Additionally, Phase I trials are evaluating drugs such as levotofisopam and Marine Active. The primary goal of these innovative therapies is to improve serum uric acid control in patients with symptomatic hyperuricemia, particularly those with comorbid conditions. These new agents aim to offer enhanced tolerability and minimize adverse events compared to traditional treatments. However, it is important to acknowledge that uricosuric agents and emerging therapies that increase renal clearance of uric acid may also raise the risk of renal adverse events.^188^

#### Lesinurad (RDEA594)

Lesinurad is a common URAT1 inhibitor that influences the serum uric acid concentration through the inhibition of URAT1 and OAT4.^394^ Lesinurad is metabolized and eliminated predominantly by the liver (75%) and to a lesser extent by the kidneys (25%), with a half-life of approximately 9 to 10 h. The primary treatment-emergent adverse events reported with its use include serious cardiovascular events and potential nephrotoxicity. Nonetheless, clinical studies have demonstrated that lesinurad at a dosage of 200 mg once daily does not elevate the risk of renal, cardiovascular, or other adverse events beyond those associated with xanthine oxidase (XO) inhibitors alone, with the exception of transient increases in serum creatinine levels.^417^ Nephrotoxicity emerges as the primary adverse effect associated with lesinurad, with its incidence being dose dependent. A recent Phase III clinical trial focused on gout patients who were intolerant or contraindicated to xanthine oxidase inhibitors (XOIs) revealed that lesinurad monotherapy at a dosage of 400 mg led to a significant incidence of elevated serum creatinine. Additionally, the trial reported renal-related adverse events, including serious adverse events, at a higher rate compared to the placebo group.^383^ However, Lesinurad is a relatively new pharmaceutical agent, currently lacks data regarding its potential impact on cardiovascular and renal outcomes.

#### Arhalofenate (MBX-102)

Arhalofenate (MBX-102) is a uricosuric compound that has been investigated for its potential in the treatment of gout and hyperuricemia. It acts as a dual-acting agent, combining the properties of a uricosuric and a nonsteroidal anti-inflammatory drug (NSAID). By inhibiting URAT1, arhalofenate promotes the excretion of uric acid in the urine, reducing serum uric acid levels.^384^ Furthermore, arhalofenate has anti-inflammatory properties, which can be beneficial in the context of gout, where inflammation plays a significant role in joint symptoms. However, clinical trials of arhalofenate have been conducted to assess its efficacy and safety in the treatment of gout and hyperuricemia. These trials aimed to evaluate its ability to lower serum uric acid levels, reduce gout flares, and provide anti-inflammatory effects.^418^ Arhalofenate is available for oral administration in daily dosages are 200, 400, or 600 mg once. The U.S. Food and Drug Administration (FDA) and the European Medicines Agency (EMA) respectively concurred with a pharmaceutical company on the efficacy endpoints for two distinct clinical indications of arhalofenate in 2017. Currently, three Phase III trials are in progress, designed to assess the comparative efficacy of a combination therapy consisting of arhalofenate plus febuxostat 40 mg versus febuxostat monotherapy at 80 mg. These trials aim to evaluate the reduction in serum uric acid levels and the incidence of gout flares.^419^

#### Dotinurad

Dotinurad represents a novel and selective urate reabsorption inhibitor that has received approval for clinical application in Japan. This medication potently inhibits the URAT1 transporter to a lesser extent, which also affects ABCG2, OAT1, and OAT3.^420^ Phase III clinical trials have substantiated the non-inferiority of dotinurad in comparison to both febuxostat and benzbromaron.^421^ Notably, dotinurad has demonstrated safety in patients with an estimated glomerular filtration rate (eGFR) between 60 ml/min and 30 ml/min, without necessitating any dosage adjustments. In terms of adverse drug reactions, aggregated data from phase II and III studies indicate that while dotinurad can potentially cause liver-related adverse reactions, such as hepatic steatosis and abnormal liver function tests, which the incidence is lower compared to benzbromarone.^420^

#### Tranilast

Tranilast, is recognized as an anti-inflammatory agent initially designed for treating allergic conditions such as asthma and allergic rhinitist.^422–424^ Recent research has shed light on its efficacy in lowering urate levels by inhibiting renal transporters URAT1 and Glucose Transporter 9 (GLUT9).^425^ In two separate preclinical trials, tranilast exhibited promising urate-lowering effects along with a reduction in inflammation associated with urate crystal deposition. Following the administration of a single dose of tranilast, a mean reduction in serum uric acid levels of 0.17 mg/dL at 4 h and 0.24 mg/dL at 24 h was observed. Headache was notably identified as the predominant adverse effect associated with tranilast. Furthermore, tranilast has been investigated in Phase II clinical trials in combination with allopurinol for patients experiencing moderate-to-severe gouty arthritis.^418^

#### Ulodesine (BCX4208)

Ulodesine, an inhibitor of purine nucleoside phosphorylase (PNP), operates upstream of xanthine oxidase (XO) in the purine metabolism pathway. By blocking PNP activity, ulodesine diminishes the substrates available for XO, thereby reducing uric acid production.^426^ Currently, this drug is in development for managing hyperuricemia in chronic gout. Promising results have been observed in two Phase II clinical trials, evaluating ulodesine both as a monotherapy and in combination with allopurinol. In a 24-week extension study, the treatment response rates for 5 mg, 10 mg, and 20 mg doses were 40%, 50%, and 45%, respectively, compared to a 25% response rate for the placebo.^427,428^ Interestingly, no significant adverse events were documented compared to the placebo group. Ulodesine displays no interactions with CYP450 isoforms and undergoes no hepatic metabolism, thereby minimizing anticipated drug interactions. However, there are concerns regarding its potential effects on T cells. Deficiency of purine nucleoside phosphorylase (PNP) has been linked to immunodeficiency and autoimmune disorders.^243^

#### Uricase

Uricase, facilitating enhanced uric acid excretion involves the administration of exogenous urate oxidase, is an enzyme which not endogenously expressed in humans. Uricases are employed in the treatment of refractory gout and are capable of achieving a rapid reduction in hyperuricemia, significant resolution of tophi, alleviation of chronic joint pain, and enhancement of overall quality of life.^387^ Urate oxidase catalyzes the breakdown of uric acid into 5-hydroxyisourate, which subsequently undergoes spontaneous degradation to allantoin without enzymatic assistance. The heightened solubility of allantoin, surpassing that of uric acid by 5-10-fold, facilitates its more efficient renal excretion.^429^ Addressing this constraint, the development of a recombinant urate oxidase, rasburicase and pegloticase has shown superior efficacy in rapidly reducing uric acid plasma concentrations compared to allopurinol.^387^ Pegloticase, a recombinant urate oxidase conjugated to polyethylene glycol (PEG), has been introduced to reduce immunogenicity and extend the half-life of rasburicase. Nevertheless, recent concerns regarding the development of antibodies against PEG in healthy blood donors prompt further exploration of potential implications for the efficacy of PEGylated pharmaceuticals. However, the main side effects include serious cardiovascular events and infusion reactions.^1^ Rasburicase is approved for managing hyperuricemia linked to chemotherapy in cancer patients. It is known for its immunogenicity and infusion-related reactions, like pegloticase. Unlike pegloticase, however, rasburicase is not PEGylated and has a shorter half-life of 8 hours.^430^ Rasburicase is predominantly employed in the management of tumor lysis syndrome owing to its rapid onset of action and short treatment duration. However, it is associated with significant incidences of infusion reactions, anaphylaxis, methemoglobinemia, and hemolysis, particularly in patients with glucose-6-phosphate dehydrogenase (G6PD) deficiency.^431^ However, the application of uricases has been constrained by factors such as availability, cost, and immunogenicity. Despite these limitations, uricases have the potential to become a primary therapeutic option for severe and challenging cases of hyperuricemia. They may be utilized as induction or debulking therapy to lower the urate pool, followed by maintenance with urate-lowering therapy (ULT).

### Therapy frame and future considerations

Per the 2020 American College of Rheumatology guidelines, allopurinol is recommended as the initial therapeutic approach for symptomatic hyperuricemia, aiming to achieve a target serum uric acid level below 6 mg/dl.^432^ Epidemiological studies suggest that maintaining a serum uric acid level below 5.5 mg/dl may offer enhanced cardiovascular and renal protection compared to target levels of 6 mg/dl or higher.^433^ Maintaining urate levels at or below 360 μmol/L through urate-lowering therapy is deemed safe and crucial for the prevention and reversal of joint, cardiovascular and renal damages.^308,362,363,372,434–436^ Additionally, EULAR guidelines recommend losartan for patients with hypertension and fenofibrate for those with hyperlipidemia, both of which have mild uricosuric effects.^336,437^ Additionally, the British Society for Rheumatology advocates for a lower target serum uric acid level of less than 5 mg/dl.^438^ Similarly, the Japanese Society of Gout and Nucleic Acid Metabolism endorses allopurinol as the primary therapy for hyperuricemia, with a target serum uric acid level of less than 6 mg/dl. Furthermore, they suggest initiating treatment for hyperuricemia when the serum uric acid level exceeds 8 mg/dl with at least one complication, or above 9 mg/dl without any complications.^439,440^ Concurrently, the development of novel uricosuric compounds aims to address resistant hyperuricemia while maintaining a favorable safety profile. The uricosuric agents targeting the URAT1 transporter, such as lesinurad and dotinurad, along with the formulation of oral combination therapies comprising xanthine oxidase inhibitors and uricosuric agents, is poised to improve the attainment of the target serum uric acid level of less than 6 mg/dL.^387^ In conclusion, navigating the intricacies of hyperuricemia treatment requires a tailored approach. To mitigate acute arthritis attacks and associated complications, early initiation of pharmacotherapeutic interventions is advisable. The dynamic landscape, characterized by established agents and novel contenders, necessitates ongoing research to optimize therapeutic strategies, ensuring efficacy while mitigating potential risks.^441^

## Conclusion and perspective

Among the history of hyperuricemia development, uric acid accumulation in serum characterizes hyperuricemia, a condition with diverse physiological roles ranging from antioxidant processes, pro-oxidative activities, pro-inflammatory activities, nitric oxide regulation, anti-aging effects and innate immune response.^14–16^ Understanding its multifaceted functions and mechanisms is crucial for deciphering its implications in health and disease. Furthermore, circulating urate levels are intricately regulated by the balance between urate production and excretion, and the kidney plays a pivotal role in maintaining this homeostasis.^186,187^ Urate transporters contribute to this delicate equilibrium. Genetic factors, particularly variations in urate transporter genes, significantly influence individual susceptibility to hyperuricemia.^245,442^ Altered urate transport mechanisms, both in the gastrointestinal tract and kidneys, are implicated in the pathogenesis of diseases associated with hyperuricemia.(Fig. 4) Future genome-wide association studies should aim to broaden their scope by encompassing diverse ethnic groups and varied patient populations. Furthermore, investigations into comorbidities linked with hyperuricemia need expansion to better elucidate the role of transporter gene mutations in disease pathogenesis.^303,443^ In the meantime, by utilizing the results of GWAS studies, clinicians can identify individuals at risk for adverse drug reactions better, thereby improving safety and treatment compliance.^150^ Furthermore, redefining hyperuricemia as a dynamic variable rather than a static biochemical parameter may offer novel perspectives on its role in disease progression. As our understanding advances, the intricate nature of hyperuricemia-related diseases necessitates ongoing exploration, emphasizing the importance of innovative personalized medicine approaches and a nuanced perspective to unravel these complexities. Dysmetabolic intestinal flora potentially contributes to gout-related metabolic and inflammatory symptoms by promoting Th17 infiltration. The identification of probiotic strains, such as DM9218, capable of lowering uric acid levels represents a novel therapeutic avenue. Currently, the modulation of intestinal microbiota through microecological therapies, including probiotics, prebiotics, and fecal microbiota transplantation, is a prominent area of clinical investigation for preventing and managing hyperuricemia and gout. These interventions aim to restore intestinal microecological balance, increasingly recognized as pivotal in the pathophysiology of these conditions. By targeting the gut microbiome, these therapies offer a novel and potentially effective strategy for mitigating the risks and progression of hyperuricemia and gout, thereby contributing to a more holistic and personalized treatment approach in clinical practice.^143,444^ Furthermore, identifying the distinct phenotypes is crucial, as hyperuricemia stemming from increased XO activity may have a different correlation with cardiovascular disease compared to that caused by renal underexcretion. Moreover, patients with the underexcretion phenotype might respond more favorably to uricosuric agents than to XO inhibitors. We suspect that biases in patient population selection could account for the inconsistent findings regarding the cardio and nephroprotective effects of hypouricemic agents in clinical trials. Differentiating between gout and asymptomatic hyperuricemia can be challenging, as gouty nephropathy may occur even in the absence of clinically apparent gout or with serum uric acid levels below the solubility threshold. There is an urgent need for future studies to delineate the effects of different classes of hypouricemic drugs on each hyperuricemia phenotypes.^372^

In addition, in terms of the major mechanism of hyperuricemia induced commodities, oxidative stress, inflammatory signaling pathway and immune response are involved in this process, which mainly lead to cell apoptosis and endothelial dysfunction.^105,270,287^(Fig. 5) While some associations have been observed, particularly with gout and renal diseases, evidence from Mendelian randomization studies did not consistently support a causal relationship between elevated serum urate levels and other metabolic or cardiovascular disorders. However, hyperuricemia plays a role in promoting inflammation, oxidative stress, and endothelial dysfunction underscores its potential contribution to disease pathogenesis.^249,445–449^ Uric acid exerts multifaceted effects on endothelial function and vascular health through its interactions with NO, ROS, and inflammatory pathways, highlighting its potential role in the pathogenesis of cardiovascular diseases like atherosclerosis.^69,90,256^ Targeting these pathways may offer therapeutic opportunities for mitigating the adverse vascular effects of hyperuricemia. However, the precise mechanisms by which NLRP3 is activated in response to monosodium urate crystals remain incompletely understood.^91,114,263,423,450,451^ The caspase-1-independent pathways of IL-1 production, including the specific proteases involved and the stimuli for their activation, are still not well-defined. Clarifying the stages at which these pathways contribute to the inflammatory phenotype and identifying the cell types orchestrating this inflammasome-independent response are crucial areas for further investigation.^337,340,452^ Moreover, a pivotal area of research concerns the mechanisms that precipitate gouty attacks in patients with sustained monosodium urate crystal deposits. It remains uncertain whether distinct initiation mechanisms trigger the inflammatory response by acting on priming signaling pathways, or if a reduction in the negative regulation of NLRP3 activation amplifies the inflammatory cascade.^15,453,454^ Current therapeutic approaches for hyperuricemia focus on mitigating associated complications and reducing serum UA levels with non-pharmacological interventions include dietary modifications and some environment factors.^455^ Besides, pharmacological interventions primarily involve urate-lowering drugs, uricosuric compounds and emerging agents.^55^ Traditional XO inhibitors and newer uricosuric compounds provide additional options for personalized treatment approaches. Newer uricosuric compounds, such as probenecid, lesinurad and arhalofenate, target different aspects of uric acid metabolism, providing additional options for personalized treatment approaches. Compared with XOIs, uricase, rasburicase and pegloticase convert UA to allantoin in adults with gout resistant to conventional therapy.^429^ Precision medicine guided by genetic insights holds promise for tailoring hyperuricemia management to individualized needs.^410^ Efforts are underway to develop novel therapies addressing unmet needs, such as alternative agents for long-term management of hyperuricemia.^201,411,438,456^ Integration of genetics into hyperuricemia research offers opportunities for advancing personalized medical approaches and improving patient outcomes. In the future, precision medicine, guided by genetic variants, represents a promising avenue for tailoring hyperuricemia management to individualized needs. Hyperuricemia continues to be inadequately managed, primarily due to factors such as ineffective dosing of urate-lowering therapy, patient noncompliance, and intolerance/adverse events associated with current treatment options.^23,321,325,415,455^ These limitations highlight the urgent needs for the creation of alternative treatment options capable of safely and effectively reducing serum uric acid levels for the long-term management of hyperuricemia. Acknowledging the growing need for better control of hyperuricemia, considerable research has been dedicated to the discovery and development of innovative therapies designed to meet these clinical challenges.
